# The impact of functional and social value on the price of goods

**DOI:** 10.1371/journal.pone.0207075

**Published:** 2018-11-12

**Authors:** Kevin Hoefman, Aaron Bramson, Koen Schoors, Jan Ryckebusch

**Affiliations:** 1 Department of Economics, Ghent University, Ghent, Belgium; 2 Howest University College West Flanders, Kortrijk, Belgium; 3 Laboratory for Symbolic Cognitive Development, Riken Brain Science Institute, Wako City, Saitama, Japan; 4 Department of Software and Information Systems, UNC Charlotte, University City Blvd., Charlotte, NC 28223, United States of America; 5 National Research University, Higher School of Economics, Moscow, Russia; 6 Department of Physics and Astronomy, Ghent University, Ghent, Belgium; Middlesex University, UNITED KINGDOM

## Abstract

According to hedonic pricing theory (HPT) market forces operate on individual characteristics of a good, and the price of a product is the aggregate of the price across those characteristics. The relationship between price and characteristics remains poorly understood because characteristic qualities are hard to quantify, people have varying levels of information about characteristics, and people have heterogeneous preferences over characteristics. By analyzing data from a large, market-driven virtual world we are able to test HPT, while largely avoiding these pitfalls. We find that a linear model with functional characteristics predicts the prices poorly, but a log-linear model performs quite well. Adding social characteristics to this log-linear model improves the predictions substantially. This work strongly supports HPT and demonstrates a “rational” calculus including social value.

## Introduction

The canonical model for the determination of the price of an economic good is that of supply and demand, where market dynamics establish an equilibrium price at which aggregate demand is equal to aggregate supply. While useful in many situations, it is difficult to apply the supply and demand model to the price differences observed among differentiated goods having multiple interacting qualities. For this reason it is necessary to move the focus of analysis from the supply and demand of goods to the supply and demand of qualities of those goods.

## Hedonic pricing theory

In a seminal paper, Rosen proposed a model called hedonic pricing theory (HPT) that describes the workings of a market for differentiated goods [1–3]. In this model, the price of a product is the aggregate of the price consumers are willing to pay for each quality characteristic of said product. As a consequence, products with more desirable characteristics command higher prices than products that are perceived to be of lower aggregate quality. HPT is used in markets such as wine [4–6], housing [7–9], art [10, 11], cars [12], and agriculture [13]. But despite this widespread use and the clear formal structure of the theory, the econometric relationship between quality and price is still not well understood due to difficulties acquiring the proper data.

While accurate information on product prices is often readily available, reliable quality information is much harder to obtain. Challenges can be summarized into six main categories. First, one would have to be able to identify all the relevant quality factors, since an underspecified model would give inaccurate predictions. Second, some quality factors (such as terroir for wine) are almost impossible to quantify. Third, consumers tend to have heterogeneous preferences, implying that what constitutes a quality factor for some may be irrelevant to the purchasing decision of others. Fourth, consumers may not only have preferences over goods’ functional qualities, but also over their social qualities such as prestige and exclusivity (see further). These are often idiosyncratic, hard to measure, or even unobservable. Fifth, consumers may have preferences over goods’ perceived social externalities such as environmentally friendly goods [14, 15], non-GMO goods [16], organic goods [17, 18], fair trade goods [19], etc. Social norms conferred via descriptive or injunctive signals [20] may lead them to believe these goods are morally superior. Recent research [21, 22] shows individual preferences may depend on the framing of available options as moral or immoral. Sixth, consumers with imperfect information may fail to correctly assess quality, either because they don’t have the necessary expertise or because the cost of obtaining the information is too high. These factors make real-world data on quality very noisy at best, rendering it difficult to pick up a pattern even if it is present. While this is unavoidable because the world is a complex place, it also means that it is difficult to test HPT (like many other economic and social theories) using real world data.

If it were possible to obtain perfect information about the quality for a range of products of a differentiated good, one could create a model to determine whether HPT explains the relationship between those qualities and prices. Identifying such a relationship would offer solid support for HPT as a scientific theory. And in doing so, it could provide strong evidence of consumer rationality [23] by demonstrating that the expenditure of resources can be strictly explained by the underlying quality of the purchased good. However, applying this degree of scientific scrutiny towards HPT has been thus far impossible.

Towards this end we analyze the less noisy environment of a virtual world called Eve Online. Virtual worlds are computer-based environments offering unique opportunities for testing economic and social theories [24, 25] due to the following properties: (1) a computer keeps track of the state of everything that populates it; (2) properties of items are precisely defined; (3) data is available about the entire population of participants; (4) all participants can be given access to all relevant details, (5) players can act in a situation of (almost) perfect information; (6) participants tend to have more homogeneous preferences over the quality characteristics because virtual worlds are invariably simpler than the real world; and (7) perceived moral qualities of a good are not expected to play any role given the general amoral nature of the environment. Although most games are too simple to provide support for such an endeavor, some large-scale virtual worlds embody dynamics that are representative of the behavior we would like to study in the real world.

## A virtual laboratory

Eve Online (EVE) is an open-ended Massively Multiplayer Online Game set in a science fiction universe created by Icelandic company CCP Games in 2003. More than 500,000 players compete for resources and territory while engaging in a variety of professions and activities including mining, manufacturing, trading, piracy, exploration, and combat, both versus the environment and against other players. As a sandbox game [26], EVE provides its players with a virtual world and the tools to explore, but players have the freedom to choose what, when, and how to approach the available content, including the purchase and sale of goods.

**Fig 1:**
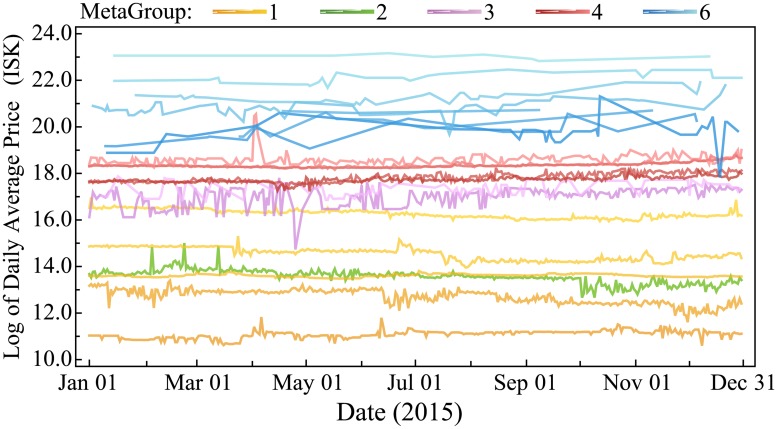
Time series of the natural log (ln) of prices in the main trading hub in 2015 for each of the 21 varieties of the module class Ballistic Control System. Module prices are subject to supply and demand dynamics but maintain an overall stable price ranking. Diagrams for other items are available in the Supplemental Information.

EVE contains 12,709 distinct items that can be bought and sold between players including ships, ship modules, minerals, ammunition, blueprints, and many more. The items available and their characteristics (which players can view at any time at zero cost) are decided by CCP and only rarely adjusted. Market prices, on the other hand, are endogenously determined by the market behavior of players via a double auction system that matches buy orders with sell orders. Prices fluctuate daily around a mostly stable base (see Fig 1). Players can buy and sell anywhere in the virtual universe, but for reasons of efficiency market activity tends to cluster in hubs. Two thirds of all market transactions are conducted in a single central trading hub.

Items and resources in the game are bought and sold with an in-game currency called the Inter-Stellar Kredit (ISK). Players earn ISK as a reward for engaging in activities such as defeating pirates, running missions, selling resources gained through mining, selling goods made through industry, offering services like courier contracts or protection to other players, or by paying real-world money. Most players spend time in the game earning ISK to purchase ships, modules, skills, etc. Advanced players even pay for their game subscription through their in-game earnings. One billion ISK is roughly equivalent to $15 USD in the period of our analysis. Because ISK has both in-game and real-world value, and because losses in EVE are permanent, players tend to be risk averse, and this fosters realistic economic behavior.

Our analysis focuses on ship modules (henceforth ‘modules’) such as weapons and defensive systems that can be fitted onto ships to improve their performance. In line with the terminology of Rosen, a module *class* contains all module varieties of the same type. There are 296 different module classes in EVE. Of these we select the 20 most traded based on total market revenue for modules in 2015 (equivalent to approximately 12 million USD, 2015 is a typical year in the Eve universe), representing 35% of total module revenue. We filter out 4 of these because the game designers changed their characteristics during 2015. The remaining 16 module classes contain between 14 and 43 varieties, for a total of 396 distinct modules in our analysis. These modules bestow 1 or 2 *benefits* (Armor, Shield, Damage, Range and/or Strength) where a *higher* value implies a better item and add between 0 and 3 *constraints* (CPU, Power and/or Energy) where a *lower* value implies a better item, depending on the class. As a consequence the total number of functional quality characteristics for each module class varies from a minimum of 1 to a maximum of 5. This heterogeneity in the number of explanatory variables prevents us from analyzing the price of all modules within a single regression.

HPT predicts that modules with higher values for the benefits should be more expensive, whereas higher values for the constraints should result in a lower price. In light of the observed price consistency (recall Fig 1), we take the mean of the average daily price in the main trading hub over 2015 as the reference price for each module. Table 1 shows the characteristics and in-game reference prices for each variety of our running example module class: it has a single benefit (Damage) and a single constraint (CPU). Characteristics and prices for all module classes can be found in the Supplemental Information. The information in these tables is available to players in the game at all times.

**Table 1:** Properties and mean of the daily average price in 2015 of all 21 varieties in the example module class.

	Module Name	Functional Qualities		Social Qualities		Price (ISK)
		Damage (+%)	CPU	MetaGroup	Rarity	
1	Ballistic Control System I	15.02	35	Tech I = 1	1	65 968
2	Cross-linked Bolt Array I	16.59	37	Tech I = 1	3	346 039
3	Ballistic Control System II	21.55	40	Tech II = 2	1	816 551
4	Muon Coil Bolt Array I	18.16	39	Tech I = 1	3	816 602
5	Multiphasic Bolt Array I	19.74	40	Tech I = 1	3	2 139 204
6	‘Pandemonium’ Ballistic Enhancement	21.33	42	Tech I = 1	3	11 818 778
7	Ballistic ‘Purge’ Targeting System I	18.25	30	Storyline = 3	4	26 122 686
8	‘Full Duplex’ Ballistic Targeting System	19.35	30	Storyline = 3	4	36 102 807
9	Domination Ballistic Control System	21.55	28	Faction = 4	3	50 382 156
10	Republic Fleet Ballistic Control System	21.55	28	Faction = 4	4	55 074 387
11	Caldari Navy Ballistic Control System	24.31	24	Faction = 4	2	92 894 448
12	Dread Guristas Ballistic Control System	24.31	24	Faction = 4	3	94 448 681
13	Khanid Navy Ballistic Control System	24.31	24	Faction = 4	4	127 593 395
14	Mizuro’s Modified Ballistic Control System	22.10	31	Officer = 6	4	435 538 765
15	Hakim’s Modified Ballistic Control System	22.66	34	Officer = 6	4	444 063 110
16	Gotan’s Modified Ballistic Control System	23.21	36	Officer = 6	4	561 835 333
17	Tobias’ Modified Ballistic Control System	23.76	39	Officer = 6	4	945 333 333
18	Kaikka’s Modified Ballistic Control System	25.00	26	Officer = 6	4	1 137 267 911
19	Thon’s Modified Ballistic Control System	25.69	29	Officer = 6	4	1 900 513 954
20	Vepas’ Modified Ballistic Control System	26.38	31	Officer = 6	4	4 023 867 247
21	Estamel’s Modified Ballistic Control System	27.07	34	Officer = 6	4	10 021 052 707

## Pricing models with functional quality

HPT is agnostic about the functional form of the relationship between price and quality [27, 28]. For example, while people who aren’t trained economists (and many who are) intuitively expect a linear relationship such that a product twice as good in overall quality would also be twice as expensive [29, 30], several empirical hedonic pricing studies have found that nonlinear forms typically perform better than the linear relationship (see [5] for an overview). It is well understood that the functional form is critical to the accuracy and consistency of the econometric model [31–33], so here we explore both a linear and non-linear relationships between price and quality.

For each of the 16 module classes in our dataset we analyze the degree to which prices within the module class are explained by the relevant functional quality characteristics using ordinary least squares regression. We also test for a log-linear relationship by using the natural logarithm of the price as the dependent variable. This process yields two sets of equations relating the price *P*_*i*_ of each module in class *i* ∈ {1, 2, …, 16} to the benefits and constraints of its 1 ≤ *n*_*i*_ ≤ 5 functional quality characteristics *F*_*ij*_:

$$\begin{array}{c} \;P_{i}=\alpha_{i}+\sum_{j=1}^{n_{i}}\beta_{ij}F_{ij}\;\left(\text{Linear}\;\text{Functional}\;\text{Quality}\right), \end{array}$$ (1)

$$\begin{array}{c} \ln P_{i}=\alpha_{i}+\sum_{j=1}^{n_{i}}\beta_{ij}F_{ij}\;\left(\text{Log}-\text{Linear}\;\text{Functional}\;\text{Quality}\right). \end{array}$$ (2)

Here, the coefficient *α*_*i*_ captures any systematic contributions to price that cannot be attributed to the quality parameters included in the model.

In the Supplementary Materials we present the goodness of fit for all 16 module classes in terms of the coefficient of determination *R*^2^ (the proportion of the variance in the predicted module price, *P*_*i*_ or ln *P*_*i*_, that is predictable from the independent variables). We also provide two alternate measures to compare the performance of competing models: the adjusted *R*^2^ and the Akaike Information Criterion (AIC), an entropy-based metric where a lower value indicates a better fit [34]. In order to assess the overall performance of the models we average the performance measures across all 16 modules. These results are summarized in Table 2.

**Table 2:** Performance of the four pricing models averaged across the 16 module classes.

Pricing model	*R*^2^	adjusted *R*^2^	AIC
linear functional quality (Eq 1)	49.6% ± 21.6%	42.5% ± 22.7%	1076.4 ± 356.8
log-linear functional quality (Eq 2)	79.5% ± 10.3%	75.9% ± 12.4%	95.8 ± 27.7
log-linear functional + social quality (Eq 3)	95.35% ± 2.77%	93.76% ± 4.12%	63.26 ± 20.47
log-linear functional + social quality (Eq 4)	95.42% ± 2.95%	93.93% ± 3.83%	61.84 ± 23.84

We find that the log-linear model captures the contribution of quality to price rather well. Averaged across all 16 modules, the log-linear model explains 79.5% of the variance in prices as measured by *R*^2^, and the coefficients of the quality characteristics consistently have the correct sign (positive for benefits, negative for constraints). The linear model doesn’t perform nearly as well, with an average *R*^2^ of 49.6% and coefficients that often have the wrong sign.

**Fig 2:**
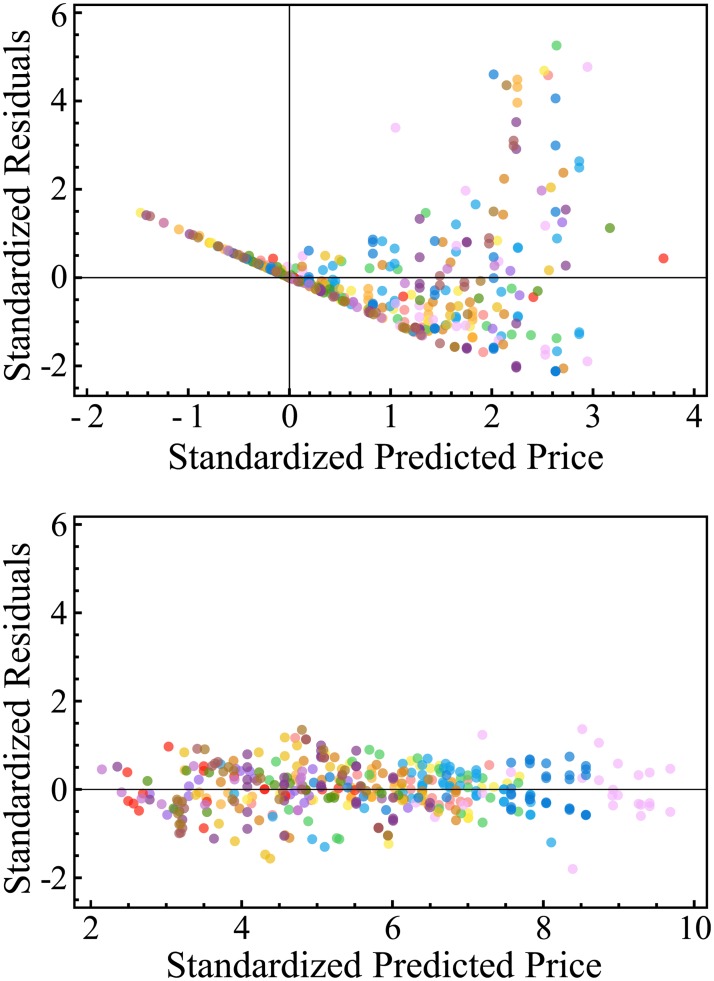
Comparison between the standardized predictions of the linear functional quality pricing model (top) and the log-linear functional quality pricing model (bottom) colored by module class (16 in total).

Fig 2 plots the standardized residuals of the fitted values versus residuals. The values are divided by the standard deviation of prices within each module class. The linear model predicts negative prices, and the residuals demonstrate uncaptured dynamics and sometimes errors larger than the fitted values themselves. The predicted values of the log-linear model, however, are in range with the actual observed prices, and the residuals are randomly distributed (meaning the model is correctly specified).

## The contribution of social value

So far our pricing models have only considered *functional quality*: those characteristics that affect the (objective) performance of a product. Our dataset includes module classes with multiple items having identical functional qualities (e.g., modules 9-10 and 11-13 in Table 1). Often, large price differences occur between these functionally identical modules; for example, players pay a surplus of 37% for module 13 over 11 for no functional benefit. Furthermore module 17 is functionally *inferior* to modules 11-13, yet its price is an order of magnitude higher. These discrepancies seem to refute the core concept of HPT. If the price of a product is the aggregate of what people are willing to pay for the individual quality factors of said product, then modules with identical quality characteristics should command the same price. That is, unless we accept that other, non-functional characteristics have value too.

The frequent occurrence of modules with identical functional properties commanding different prices in our dataset allows us to isolate what has been termed “conspicuous consumption” in the literature. The willingness to pay extra for no discernible benefit was first described by Veblen [35] in 1899 as a way for the rich to demonstrate their wealth. Whether it is rational to pay for no added functional benefit is a controversial topic in economics even today. The more classical explanation is one of competition for status goods via the expenditure of wealth [36, 37] which can lead to negative consumption effects [38, 39]. Interestingly, it is often those earning the least that spend the greatest fraction of their income on conspicuous consumption [40, 41]. The motivation for engaging in conspicuous consumption is classically explained by the comparative advantages obtained from (increased) social status, such as sexual selection [42]. But more recent research reveals that psychological aspects like identity [43], social belonging [44] and self-esteem [45, 46] also play a role. We use the term *social value* to refer to benefits that are derived from the social confirmation of a consumer’s ability to pay. Since social value exists in the interaction with other people, only characteristics that can be observed by others are candidates for this type of consumption. In EVE all modules share two characteristics that are observable by all players yet add no functional benefit to the module.

The first social variable is the Rarity of the module, which can be observed by looking at market volumes. Any positive effect of rarity on prices within a class of differentiated products cannot be attributed to the dynamics of supply and demand alone. When two products are identical in quality yet different in price, rational buyers should always prefer the cheaper alternative. This would cause demand to shift to the cheaper substitute, until an equilibrium is reached where both prices are the same even though their supply may be different. Economists refer to this as the non-arbitrage condition of any economic equilibrium [47]. If rarity is found significant for the price while controlling for functional quality, it implies that item’s rarity confers social value to its owner.

The second social variable, the module’s so-called MetaGroup, appears as a color-coded marker on items informing players about how the item enters the game. There are six categories of MetaGroup with increasing exclusivity (*MG*_*i*_): Tech I (for the most basic modules), Tech II, Storyline, Faction, Deadspace, and Officer (the most exclusive). The meta groups are correlated with functional quality by design (see Table 1); however, this correlation varies considerably across characteristics and module classes, and significant functional quality overlaps occur across metagroups. Specifically, we even have 39 pairs of modules from different metagroups with identical functional characteristics, thus creating a natural experiment for the role of social value.

By analyzing price differences for these functionally identical pairs we find that people pay at least 2 times, and on average 7 times, more for an identical module in a higher MetaGroup. This price difference can only be understood as a social value premium: players pay for the social status of an item in addition to (or apart from) how good the item actually is in terms of functional quality.

## Pricing models with functional and social quality

Here we test the importance of the social variables along with the functional ones using econometric models of HPT.

To do this we incorporate the social quality characteristics in our pricing model by adding variables that capture their rarity and metagroup membership. To measure the contribution of rarity, we define the variable Rarity *R*_*i*_ using a logarithmic binning technique based on its average daily market volume (*v*) during 2015. Modules with the lowest rarity (i.e., *v* ≥ 10000) are assigned *R* = 0, 10000 > *v* ≥ 1000 → *R* = 1, 1000 > *v* ≥ 100 → *R* = 2, 100 > *v* ≥ 10 → *R* = 3, and *v* < 10 → *R* = 4. We measure the contribution of MetaGroup *MG*_*i*_ with a discrete variable ranging from 1 (Tech I) to 6 (Officer) of increasing exclusivity as described above.

Adding parameters *R*_*i*_ and *MG*_*i*_ to our purely functional log-linear model (2), we obtain the log-linear social pricing model in Eq 3. We test the robustness of this log-linear model with an alternative model where the contribution of each MetaGroup value is measured with a separate dummy variable *MG*_*ik*_ (except for the lowest category which is captured by the regression constant *α*_*i*_). This alternative model is shown in Eq 4.

$$\begin{array}{c} \left(\text{Log}-\text{linear}\;\text{Functional}+\text{Social}\;\text{Quality},\text{v}1\right)\\ \ln P_{i}=\alpha_{i}+\sum_{j=1}^{n_{i}}\beta_{ij}\;F_{ij}+\gamma_{i}\;R_{i}+\delta_{i}\;MG{}_{i}\;, \end{array}$$ (3)

$$\begin{array}{c} \left(\text{Log}-\text{linear}\;\text{Functional}+\text{Social}\;\text{Quality},\text{v}2\right)\\ \ln P_{i}=\alpha_{i}+\sum_{j=1}^{n_{i}}\beta_{ij}\;F_{ij}+\gamma_{i}\;R_{i}+\sum_{k=1}^{6}\delta_{ik}\;MG{}_{ik}\;. \end{array}$$ (4)

We find that the pricing model from Eq 3 that accounts for both functional value and social value explains 95.35% ± 2.77% of the observed price variance across all 16 module classes compared to 79.5% ± 10.3% for the purely functional models (see Table 2).

**Fig 3:**
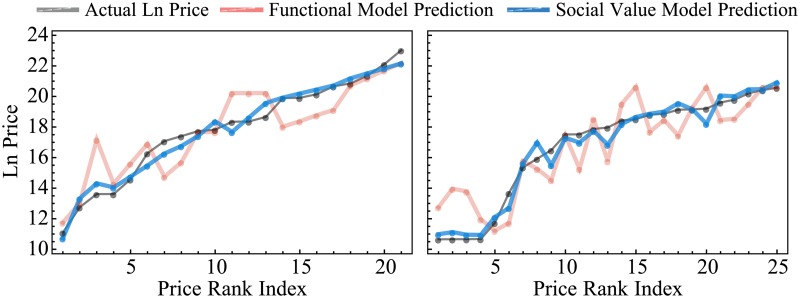
Comparison between observed prices (grey), predicted prices from the log-linear model including only functional value (red, Eq 2) and predicted prices from the log-linear model also including social value (blue, Eq 3). Left: prices from the Ballistic Control System class shown in Table 1 increase exponentially (here drawn in their natural logarithm), and this is captured accurately by the log-linear model including social value. Right: the 25 modules in the Large Shield Booster class exhibit flat prices till module 4, a sharp rise in prices between modules 4 and 8, and a smooth exponential rise from module 10 onwards. The log-linear model including social value captures these features relatively accurately.

**Fig 4:**
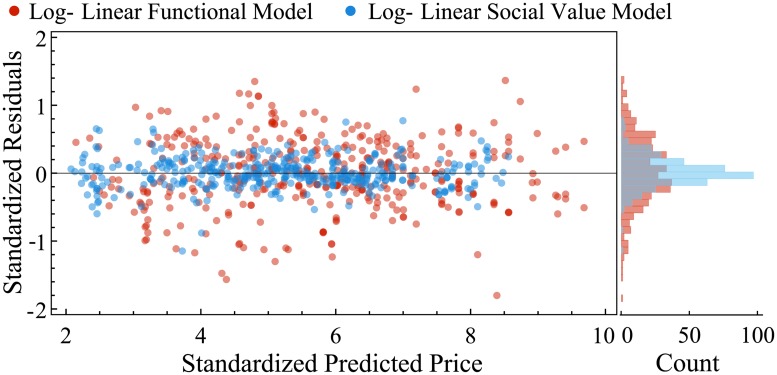
Comparison of the purely functional and social value log-linear models. Residuals of the log-linear model with (Eq 3) and without (Eq 2) social value parameters across all modules. The predicted values and residuals are standardized by dividing by the standard deviation within each module class. The addition of the social quality parameters clearly improves the accuracy of the model. On the right hand side we plot the full distribution of the standardized residuals of both models.

Adjusted *R*^2^ and AIC (from 95.8 to 63.26) confirm that the addition of the social value parameters improves the performance of the model considerably. The results from the model in Eq 4 are nearly identical. Fig 3 illustrates this by comparing the fitted values of the log-linear model with the actual price observations for two example module classes. Table 2 summarizes the results while individual regression results for each module class are shown in the Supplementary Materials. Fig 4 shows the improvement in fit for the log-linear model across all modules and module classes by adding social quality to the model by plotting their normalized residuals.

## Isolating social quality

Recall that in an HPT regression, the total price of an item is broken down into the contributions of the parameters that are included in the model. In Eq 3 (where *MG*_*i*_ is represented by a discrete variable) the effects of Rarity and MetaGroup on the total price can be isolated with 95% confidence in 8 of the 16 regressions. For these 8 module classes we employ the estimated coefficients to derive the individual contributions from each of the functional characteristics, rarity and the meta group, to the (natural log of the) predicted module price. For the model in Eq 4 we use the averages of the estimated coefficients for each of the MetaGroup values.

**Fig 5:**
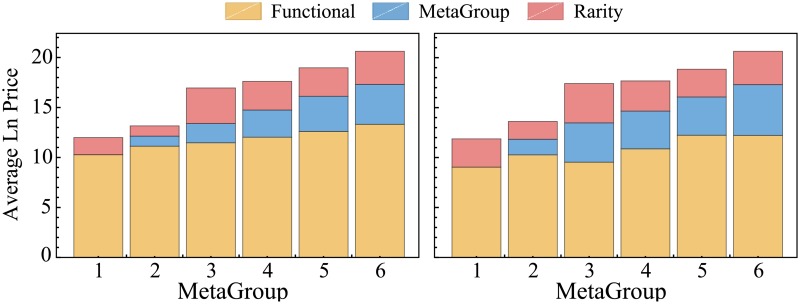
Contribution of the functional value (*F*_*ij*_) and the social value (rarity *R*_*i*_ and meta group *MG*_*i*_) to the (natural log of the) predicted price, organized per MetaGroup. Left: contribution according to the model in Eq 3. Right: contribution according to the model in Eq 4. Red and blue are the contributions of *R*_*i*_ and *MG*_*i*_ based on the estimated coefficients for these social quality parameters. Yellow is the part of the predicted module price that is unexplained by the social quality characteristics *R*_*i*_ and *MG*_*i*_ and is therefore attributed to functional characteristics *F*_*ij*_.

From Fig 5 it is clear that not only does social value contribute substantially to prices, but also that the relative contribution of social value to the price tends to rise with the more exclusive items (as should be expected). This provides clear support for the claim that social value is economically relevant and demonstrates that the economic value of these social qualities can be assessed through the lens of HPT.

## Conclusions

The question of whether Hedonic Pricing Theory can predict the prices of items within a class of differentiated goods is ultimately about consumer rationality. In previous studies imperfect information, market failures, heterogeneous preferences, and other factors have complicated the proper empirical testing of HPT. We circumvent these shortcomings by analyzing economic data from a very rich and market-driven virtual world.

Our analysis reveals that consumers appraise the social quality of a product in the same general way as its functional quality: incremental increases in the social quality of a product lead to exponential increases in the price consumers are willing to pay for the good embodying those qualities. The extremely good fit of the log-linear social quality pricing model provides strong support for HPT as an explanation for the prices of products within a class of differentiated goods.

This has important implications for economic theory and practice. Economy theory suggests that firm investments in branding in part signal the firm’s willingness to engage in a long term relationship with its clients and therefore in equilibrium reduces the information asymmetry about the product’s quality on the part of the consumers, rendering them more willing to pay a price mark-up. This argument has lead observers to predict that the ongoing digital revolution, by reducing information asymmetries between firms and consumers, would erode the value of brands and shrink price mark-ups. We show that social value considerations make consumers value labels even if they have incentives to behave rationally and their information is complete. This may inform further theoretical advances on the deeper drivers of social value and suggests that top brands’ price mark-ups may well survive the digital revolution, because their value hinges not only on the presence of information asymmetries, but also on social value.

Finally we may also conclude that consumers, if equipped with the proper information and incentives, tend to be very rational in their price decisions, as long as we properly account for social quality in addition to the purely functional quality of a product. After all, humans are social animals, so it should come as no surprise that social value is real value, too.

## Supporting information

S1 File Supplementary text. The full dataset used and additional details of our analysis results (including Fig S1 and reference 48).
(PDF)

## References

[1] Rosen S. Hedonic Prices and Implicit Markets: Product Differentiation in Pure Competition. Journal of Political Economy. 1974;82(1):34–55. 10.1086/260169

[2] Lancaster KJ. A new approach to consumer theory. Journal of political economy. 1966;74(2):132–157. 10.1086/259131

[3] Court AT. Hedonic price indexes with automotive examples. The Dynamics of Automobile Demand. 1939; p. 98–119.

[4] Oczkowski E. A Hedonic Price Function for Australian Premium Table Wine. Australian Journal of Agricultural and Resource Economics. 1994;38(1):93–110. 10.1111/j.1467-8489.1994.tb00721.x

[5] Orrego M, Defrancesco E, Gennari A. The Wine Hedonic Price Models in the ‘Old and New World’: State of the Art. Revista de la Facultad de Ciencias Agrarias. 2012;44(1):205–220.

[6] Dimsom E, Rousseau PL, Spaenjers C. The Price of Wine. Journal of Financial Economics. 2015;118(2):431–449. 10.1016/j.jfineco.2015.08.005

[7] Goodman AC. Hedonic Prices, Price Indices and Housing Markets. Journal of Urban Economics. 1978;5:471–484. 10.1016/0094-1190(78)90004-9

[8] Malpezzi S. Hedonic pricing models: a selective and applied review. Housing economics and public policy. 2002; p. 67–89. 10.1002/9780470690680.ch5

[9] Sander H, Polasky S. The value of views and open space: Estimates from a hedonic pricing model for Ramsey County, Minnesota, USA. Land Use Policy. 2009;26(3):837–845. 10.1016/j.landusepol.2008.10.009

[10] Gérard-Varet L. On pricing the priceless: Comments on the economics of the visual art market. European Economic Review. 1995;39:509–518. 10.1016/0014-2921(94)00057-7

[11] Renneboog L, Spaenjers C. Buying Beauty: On Prices and Returns in the Art Market. Management Science. 2013;59(1):205–220. 10.1287/mnsc.1120.1580

[12] Andersson H. The Value of Safety as Revealed in the Swedish Car Market: An Application of the Hedonic Pricing Approach. Journal of Risk and Uncertainty. 2005;30(3):211–239. 10.1007/s11166-005-1154-1

[13] Ready RC, Abdalla CW. The Amenity and Disamenity Impacts of Agriculture: Estimates from a Hedonic Pricing Model. American Journal of Agricultural Economics. 2005;87(2):314–326. 10.1111/j.1467-8276.2005.00724.x

[14] Kalafatis SP, Pollard M, East R, Tsogas MH. Green marketing and Ajzen’s theory of planned behaviour: a cross-market examination. Journal of consumer marketing. 1999;16(5):441–460. 10.1108/07363769910289550

[15] Cronin JJ, Smith JS, Gleim MR, Ramirez E, Martinez JD. Green marketing strategies: an examination of stakeholders and the opportunities they present. Journal of the Academy of Marketing Science. 2011;39(1):158–174. 10.1007/s11747-010-0227-0

[16] Baker GA, Burnham TA. Consumer response to genetically modified foods: market segment analysis and implications for producers and policy makers. Journal of Agricultural and Resource Economics. 2001; p. 387–403.

[17] Yiridoe EK, Bonti-Ankomah S, Martin RC. Comparison of consumer perceptions and preference toward organic versus conventionally produced foods: A review and update of the literature. Renewable Agriculture and Food Systems. 2005;20(4):193–205. 10.1079/RAF2005113

[18] Lee WcJ, Shimizu M, Kniffin KM, Wansink B. You taste what you see: Do organic labels bias taste perceptions? Food Quality and Preference. 2013;29(1):33–39. 10.1016/j.foodqual.2013.01.010

[19] Hainmueller J, Hiscox MJ, Sequeira S. Consumer demand for fair trade: Evidence from a multistore field experiment. Review of Economics and Statistics. 2015;97(2):242–256. 10.1162/REST_a_00467

[20] Schultz PW, Nolan JM, Cialdini RB, Goldstein NJ, Griskevicius V. The constructive, destructive, and reconstructive power of social norms. Psychological science. 2007;18(5):429–434. 10.1111/j.1467-9280.2007.01917.x 17576283

[21] Capraro V, Rand DG. Do the Right Thing: Experimental evidence that preferences for moral behavior, rather than equity or efficiency per se, drive human prosociality. Judgment and Decision Making. 2018;13(1):99–111.

[22] Tappin BM, Capraro V. Doing good vs. avoiding bad in prosocial choice: A refined test and extension of the morality preference hypothesis. Journal of Experimental Social Psychology. 2018;79:64–70. 10.1016/j.jesp.2018.06.005

[23] Simon H. Rationality in Psychology and Economics. The Journal of Business. 1986;59(4):209–224. 10.1086/296363

[24] Castronova E. The Price of Bodies: A Hedonic Pricing Model of Avatar Attributes in a Synthetic World. Kyklos. 2004;57(2):173–196. 10.1111/j.0023-5962.2004.00249.x

[25] Thurner S, Szell M, Sinatra R. Emergence of Good Conduct, Scaling and Zipf Laws in Human Behavioral Sequences in an Online World. PLOS ONE. 2012;7(1). 10.1371/journal.pone.0029796 PMC3257232 22253784

[26] Squire K. Open-ended video games: A model for developing learning for the interactive age. The ecology of games: Connecting youth, games, and learning. 2008; p. 167–198.

[27] Halvorsen R, Pollakowski H. Choice of Functional Form for Hedonic Price Equations. Journal of Urban Economics. 1981;10:37–49. 10.1016/0094-1190(81)90021-8

[28] Atkinson S, Halvorsen R. A New Hedonic Technique for Estimating Attribute Demand: An Application to the Demand for Automobile Fuel Efficiency. The Review of Economics and Statistics. 1984;66(3):417–426. 10.2307/1924997

[29] Karelaia N, Hogarth R. Determinants of Linear Judgment: A Meta-Analysis of Lens Model Studies. Psychological Bulletin. 2008;134(3):404–426. 10.1037/0033-2909.134.3.404 18444703

[30] Deane D, Hammond K, Summers D. Acquisition and Application of Knowledge in Complex Inference Tasks. Journal of Experimental Psychology. 1972;92(1):20–26. 10.1037/h0032162

[31] Halvorsen R. On the Choice of Functional Form for Hedonic Price Functions. The Review of Economics and Statistics. 1988;70(4):668–675. 10.2307/1935831

[32] Brown J, Ethridge D. Functional form model specification: An application to hedonic pricing. Agricultural and Resource Economics Review. 1995;24(2):166–173. 10.1017/S1068280500008807

[33] Triplett J. Handbook on Hedonic Indexes and Quality Adjustments in Price Indexes. OECD Publishing; 2004.

[34] Bozdogan H. Model selection and Akaike’s Information Criterion (AIC): The general theory and its analytical extensions. Psychometrika. 1987;52(3):345–370. 10.1007/BF02294361

[35] Veblen T. The theory of the leisure class: An economic study of institutions. Unwin Books; 1899.

[36] Ireland NJ. On limiting the market for status signals. Journal of Public Economics. 1994;53:91–110. 10.1016/0047-2727(94)90015-9

[37] Bagwell LS, Bernheim BD. Veblen Effects in a Theory of Conspicuous Consumption. The American Economic Review. 1996;86(3):349–373.

[38] Frank RH. The Demand for Unobservable and Other Nonpositional Goods. The American Economic Review. 1985;75(1):101–116.

[39] Hopkins E, Kornienko T. Running to Keep in the Same Place: Consumer Choice as a Game of Status. The American Economic Review. 2004;94(4):1085–1107. 10.1257/0002828042002705

[40] Charles K, Hurst E, Roussanov N. Conspicuous Consumption and Race. The Quarterly Journal of Economics. 2009;124(2):425–467. 10.1162/qjec.2009.124.2.425

[41] Kaus W. Conspicuous consumption and “race”: Evidence from South Africa. Journal of Development Economics. 2013;100(1):63–73. 10.1016/j.jdeveco.2012.07.004

[42] Sundie JM, Kenrick DT, Griskevicius V, Tybur JM, Vohs KD, Beal DJ. Peacocks, Porsches, and Thorstein Veblen: Conspicuous consumption as a sexual signaling system. Journal of personality and social psychology. 2011;100(4):664 10.1037/a0021669 21038972

[43] Akerlof GA, Kranton RE. Economics and Identity. The Quarterly Journal of Economics. 2000;115(3):715–753. 10.1162/003355300554881

[44] Han Y, Nunes J, Drèze X. Signaling status with luxury goods: The role of Brand Prominence. Journal of Marketing. 2010;74(4):15–30. 10.1509/jmkg.74.4.15

[45] Sivanathan N, Pettit N. Protecting the self through consumption: Status goods as affirmational commodities. Journal of Experimental Social Psychology. 2010;46(3):564–570. 10.1016/j.jesp.2010.01.006

[46] Bursztyn L, Ferman B, Fiorin S, Kanz M, Rao G. Status goods: experimental evidence from platinum credit cards. The World Bank; 2017.

[47] Werner J. Arbitrage and the existence of competitive equilibrium. Econometrica: Journal of the Econometric Society. 1987; p. 1403–1418. 10.2307/1913563

