# Supplementary material for: The impact of functional and social value on the price of goods
Source: PLoS One. 2018 Nov 12;13(11):e0207075. doi: 10.1371/journal.pone.0207075 (PMC6231626; doi:10.1371/journal.pone.0207075)
Supplement: S1 File — The full dataset used and additional details of our analysis results (including Fig S1 and reference 48). (PDF) [file pone.0207075.s001.pdf]

The impact of functional and social value on the price of goods – Supplemental Information

Kevin Hoefman [1 2]   Aaron Bramson [3 1 4]   Koen Schoors [1 5 *]
Jan Ryckebusch [6]

*To whom correspondence should be addressed; E-mail: koen.schoors@ugent.be.

Contents:

1. Supplementary Text (including figure S1 and reference 48)
2. Tables S1 to S17
3. Figs. S2 to S36

[1]Department of Economics, Ghent University, Tweekerkenstraat 2, 9000 Ghent, Belgium
[2]Howest University College West Flanders, Marksesteenweg 58, 8500 Kortrijk, Belgium
[3]Laboratory for Symbolic Cognitive Development, Riken Brain Science Institute, 2-1 Hirosawa, Wako City, Saitama 351-0198, Japan
[4]Department of Software and Information Systems, UNC Charlotte, Woodward 341, 9201 University City Blvd., Charlotte, NC 28223, USA
[5]National Research University, Higher School of Economics, 20 Myasnitskaya ulitsa, Moscow 101000, Russia
[6]Department of Physics and Astronomy, Ghent University, Proeftuinstraat 86, 9000 Ghent, Belgium

1 Supplementary Text

Eve Online (EVE) is an open-ended Massively Multiplayer Online Game (MMOG) developed by CCP Games that was released in North America and Europe in May 2003, and now has subscribers worldwide. It is a player-driven, persistent-world set in a futuristic galaxy, a network of 7,930 star systems connected by means of stargates and portals. The star systems can contain moons, planets, stations, wormholes, asteroid belts and complexes which drive a number of in-game activities including mining, manufacturing, trading, exploration, piracy, and combat, both against the environment and against other players. In February 2013, EVE Online reached over 500,000 subscribers (*48*).

Eve Online is an open-ended video game, or "sandbox" game (2). EVE provides its players with a virtual world and the tools to explore it, but players have the freedom to choose what, when, and how to approach the available content.

Within the universe are solar systems and regions which can be mined for minerals to craft items. EVE contains 12,709 distinct items that can be bought and sold between players including ships, ship modules, minerals, ammunition, blueprints, and many more. These items and resources can be bought and sold on the in-game marketplace using the in-game currency, the Inter-Stellar Kredit or ISK. Market prices are endogenously determined by the market behavior of players via a double auction system that matches buy orders with sell orders. Prices fluctuate daily around a mostly stable base. Players can buy and sell anywhere in the virtual universe, but for reasons of efficiency market activity tends to cluster in hubs. Two thirds of all market transactions are conducted in a single central trading hub.

The existence of the market allows players to choose their play style according to their personal preferences. Some players will prefer to manufacture goods themselves. Players who don't can purchase the what they need from others. The market finds an equilibrium among play styles. For example, if fewer players choose to spend their playtime producing goods for others then the market supply of these goods will drop. This in turn will increase prices, which in turn will entice some players to engage in production.

Resources are limited in the real world. Scarcity of resources underlies the dynamics of the real economy: where seemingly unlimited wants meet limited resources. In contrast, a computer game has the potential to provide its players with unlimited game resources. In EVE, players pay for access to the game world and to the character they play, by method of monthly subscription. A player starts out with basic skills and a basic ship. To buy more skills, better space ships, etc. players need to earn in-game money. This artificial restriction drives the economy of the game: players are denied access to advanced features of the game until they unlock these features by spending the (virtual) money of the game. This money can be earned in the game through effort on the player's part.

Players earn in-game money (called "Inter-Stellar Kredit" or ISK) as a reward for engaging in activities such as defeating pirates, running missions, selling resources gained through mining, selling goods made through industry, offering services like courier contracts or protection to other players, or by paying real-world money. The game charges ISK in return for certain

perks, most notably the acquiring of new player skills. This is where ISK ultimately derives its value. ISK is the only "good" in the Eve universe that doesn't need to be transported; players can access their account and make transactions from anywhere in the galaxy. New ISK enters the game when players engage in activities that are rewarded by the game with a payment of ISK. The sources of new ISK are called faucets. Money exits the game when players are charged ISK by the game in exchange for certain benefits. These are called sinks. The largest ISK faucet in Eve is Bounty Prices which players receive for defeating (non-player) pirates.The largest ISK sink in Eve are the skills that players need to buy before they can use most items in the game. An imbalance between ISK generation and ISK removal (for example when players create more isk through faucets than is being taken out of the game through sinks) will affect prices and the money supply.

1.1 Risks

Eve Online is considered to have a high "death penalty" compared to other games. In MMO's, the concept of death penalty covers the consequences of being killed in the game. Many games are very forgiving, as players generally don't enjoy being punished for making a mistake. In World of Warcraft, for example, characters that die will respawn (= return to the game) without any consequences other than losing a couple of minutes of game time. In EVE, when your ship is destroyed, it is permanently gone, along with much of your cargo. The resulting attrition of ships and modules creates a constant need for industrialists to produce new ships and new modules. This cycle of production and destruction is in a large part what drives the economy of Eve Online.

Another risk which only exists in the conquerable space of nullsec are the consequences of the loss of territory. Stations in conquerable space are only accessible to the current owners of that space. When a station system is conquered, players of the alliance that lost the system lose access to anything they have stored inside the station. Often, such an event goes accompanied by fire sales, and the players who are about to lose access to their goods sell these at considerably lowered prices.

1.2 Modules

In EVE all modules share two characteristics that are observable by all players yet add no functional benefit to the module. The first is the rarity of the module, which can be observed by looking at market volumes. Any positive effect of rarity on prices within a class of differentiated products cannot be attributed to the dynamics of supply and demand alone. When two products are identical in quality yet different in price, rational buyers should always prefer the cheaper alternative. This would cause demand to shift to the cheaper substitute, until an equilibrium is reached where both prices are the same even though their supply may be different. Economists refer to this as the non-arbitrage condition of any economic equilibrium (*38*). If rarity is found

significant for the price while controlling for functional quality, it implies that item's rarity confers non-functional value to its owner, which we include as broadly construed social value.

The second social variable appears as a color-coded marker informing players where each module fits in the overall quality hierarchy within a module class (see Figure S1). The quality spectrum is subdivided into six ranges of so-called meta groups: Tech I (for the most basic modules), Tech II, Storyline, Faction, Deadspace, and Officer (the most exclusive).

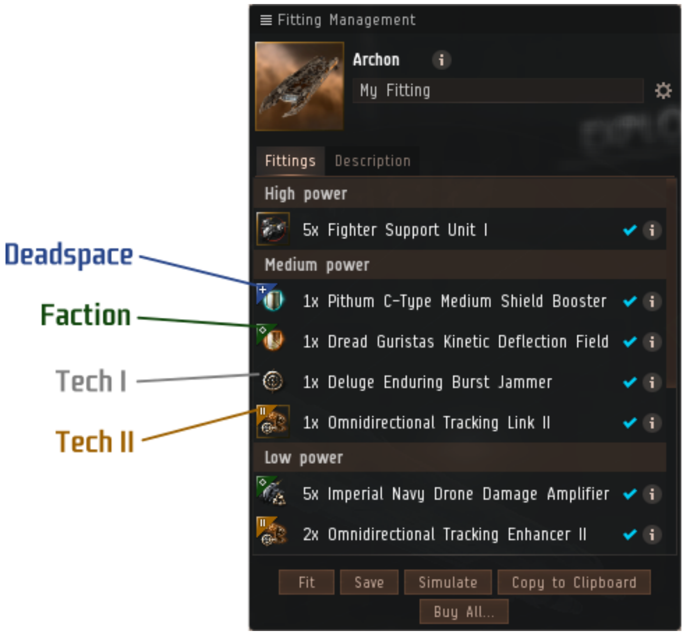

Figure S1: How meta groups values of modules appear to players in the game.

The meta groups are highly correlated with functional quality by design (e.g., see Table S4). However, there is a large amount of heterogeneity in our dataset. Significant overlap of functional characteristics occurs between neighboring meta groups, meaning modules from different meta groups can have comparable functional characteristics. Storyline modules even tend to have slightly worse functional characteristics than the lower Tech II modules. And the two most exclusive groups (Deadspace and Officer modules) are designed to overlap in terms of functional quality. This choice by the developers of the game has created what amounts to a natural experiment, whereby 39 pairs of modules exhibit identical functional qualities despite different social categories.

Considering rational consumers and market equilibrium one would expect comparable market prices for these modules of identical functional quality. Yet the difference in price is huge, and systematically in favor of the top Officer category: on average players pay 7 times more for the top Officer module, with a minimum of 2 times more. This price difference can only be understood as a social value premium: players pay for the social status of an item in addition to (or apart from) how good the item is in terms of functional quality.

1.3 Data

All the data that used in the estimations are in Tables S1 to S17. These tables can be used for reproduction. We derived these data ourselves from external websites. The historic price data and volumes are made available the CCP itself on https://eve-marketdata.com/. The data on the functional qualities of the items in the module classes we study can be found in https://www.evemarkethelper.net/MarketBrowser/9-ship-equipment and https://everef.net/categories/7. Alternatively opening a free trial account also gives immediate and free access to all data on functional qualities

References

48. Keza MacDonald, *EVE Online Passes Half a Million Subscribers*. Feb 28, 2013, http://www.ign.com/articles/2013/02/28/eve-online-passes-half-a-million-subscribers

2 Additional Tables

ID	Module Class	Benefits		Constraints			Variants
1	Adaptive Nano Plating	Armor					36
2	Energized Adaptive Nano Membrane	Armor		CPU			35
3	Ballistic Control System	Damage		CPU			21
4	Magnetic Field Stabilizer	Damage		CPU			14
5	Heat Sink	Damage		CPU			25
6	Gyrostabilizer	Damage		CPU			18
7	Shield Boost Amplifier	Shield		CPU			25
8	Adaptive Invulnerability Field	Shield		CPU		Capacitor	17
9	Armor Explosive Hardener	Armor		CPU		Capacitor	39
10	Large Armor Repair Unit	Armor		CPU	Power	Capacitor	43
11	X-Large Shield Booster	Shield		CPU	Power	Capacitor	25
12	Large Shield Booster	Shield		CPU	Power	Capacitor	25
13	Medium Shield Booster	Shield		CPU	Power	Capacitor	17
14	Stasis Webifier	Range	Strength	CPU	Power		18
15	Warp Disruptor	Range	Strength	CPU	Power	Capacitor	19
16	Warp Scrambler	Range	Strength	CPU	Power	Capacitor	19

Table S1: Overview of the 16 classes of ship modules included in our analysis along with the attributes each effects as benefits and constraints. You can see there is heterogeneity both in the number of attributes a pricing model must fit as well as in the number of variants for each module class.

Module Name	MetaGroup	Rarity	Armor	Price (ISK)
‘Collateral’ Adaptive Nano Plating I	1	2	13.5	5 143
Limited Adaptive Nano Plating I	1	3	11.7	5 300
Upgraded Adaptive Nano Plating I	1	2	9.84	5 710
Adaptive Nano Plating I	1	2	8	16 790
‘Refuge’ Adaptive Nano Plating I	1	2	15.4	48 820
Ammatar Navy Adaptive Nano Plating	4	4	17.5	150 002
Adaptive Nano Plating II	2	1	15.4	177 692
Shadow Serpentis Adaptive Nano Plating	4	3	15.4	1 244 437
Domination Adaptive Nano Plating	4	4	15.4	2 125 195
Khanid Navy Adaptive Nano Plating	4	4	17.5	2 251 605
Republic Fleet Adaptive Nano Plating	4	4	15.4	5 579 607
Dark Blood Adaptive Nano Plating	4	3	17.5	7 508 965
True Sansha Adaptive Nano Plating	4	3	17.5	7 881 240
Imperial Navy Adaptive Nano Plating	4	4	17.5	13 192 473
Federation Navy Adaptive Nano Plating	4	4	15.4	15 010 289
Corpii C-Type Adaptive Nano Plating	5	4	19.6	33 387 125
Coreli C-Type Adaptive Nano Plating	5	3	19.6	34 473 307
Centii C-Type Adaptive Nano Plating	5	4	19.6	38 535 486
Coreli B-Type Adaptive Nano Plating	5	4	21.6	44 501 752
Centii B-Type Adaptive Nano Plating	5	4	21.6	48 625 102
Corpii B-Type Adaptive Nano Plating	5	4	21.6	52 658 281
Coreli A-Type Adaptive Nano Plating	5	3	23.7	63 783 918
Centii A-Type Adaptive Nano Plating	5	3	23.7	68 118 653
Corpii A-Type Adaptive Nano Plating	5	3	23.7	76 270 475
Brokara’s Modified Adaptive Nano Plating	6	4	19.6	330 615 385
Brynn’s Modified Adaptive Nano Plating	6	4	19.6	358 939 506
Setele’s Modified Adaptive Nano Plating	6	4	23.7	376 250 002
Mizuro’s Modified Adaptive Nano Plating	6	4	19.6	546 823 394
Selynne’s Modified Adaptive Nano Plating	6	4	21.6	797 582 118
Raysere’s Modified Adaptive Nano Plating	6	4	21.6	801 200 001
Tairei’s Modified Adaptive Nano Plating	6	4	19.6	818 488 507
Cormack’s Modified Adaptive Nano Plating	6	4	25.8	938 999 906
Chelm’s Modified Adaptive Nano Plating	6	4	25.8	1 335 583 344
Ahremen’s Modified Adaptive Nano Plating	6	4	23.7	1 611 751 707
Vizan’s Modified Adaptive Nano Plating	6	4	23.7	1 802 385 525
Tuvan’s Modified Adaptive Nano Plating	6	4	21.6	2 646 500 000

Table S2: Adaptive Nano Plating module class data. Functional characteristics for each variant of the module class and average daily price for the item in the Forge region during 2015.

Module Name	MetaGroup	Rarity	Armor	CPU	Price (ISK)
Limited Energized Adaptive Nano Membrane I	1	2	15	27	65 322
Upgraded Energized Adaptive Nano Membrane I	1	2	15	28	73 801
Experimental Energized Adaptive Nano Membrane I	1	2	15	25	78 759
Energized Adaptive Nano Membrane I	1	2	15	30	207 111
Prototype Energized Adaptive Nano Membrane I	1	2	15	24	254 719
Energized Adaptive Nano Membrane II	2	1	20	36	554 561
Shadow Serpentis Energized Adaptive Nano Membrane	4	3	20	30	13 581 622
Federation Navy Energized Adaptive Nano Membrane	4	3	20	30	18 569 333
Imperial Navy Energized Adaptive Nano Membrane	4	2	22.5	30	38 040 463
True Sansha Energized Adaptive Nano Membrane	4	3	22.5	30	40 627 626
Dark Blood Energized Adaptive Nano Membrane	4	3	22.5	30	40 898 248
Ammatar Navy Energized Adaptive Nano Membrane	4	4	22.5	30	74 093 828
Khanid Navy Energized Adaptive Nano Membrane	4	4	22.5	30	86 749 695
‘Pilfer’ Energized Adaptive Nano Membrane I	3	4	20	24	92 616 647
Corelum C-Type Energized Adaptive Nano Membrane	5	3	24.4	33	104 090 054
Corpum C-Type Energized Adaptive Nano Membrane	5	3	24.4	33	105 887 582
Centum C-Type Energized Adaptive Nano Membrane	5	3	24.4	33	106 904 352
Corelum B-Type Energized Adaptive Nano Membrane	5	4	26.4	36	271 772 735
Corpum B-Type Energized Adaptive Nano Membrane	5	4	26.4	36	274 794 326
Centum B-Type Energized Adaptive Nano Membrane	5	4	26.4	36	285 594 746
Brynn’s Modified Energized Adaptive Nano Membrane	6	4	24.4	33	438 323 512
Selynne’s Modified Energized Adaptive Nano Membrane	6	4	26.4	36	574 749 750
Tairei’s Modified Energized Adaptive Nano Membrane	6	4	24.4	33	603 289 439
Centum A-Type Energized Adaptive Nano Membrane	5	4	28.3	38	739 343 777
Corelum A-Type Energized Adaptive Nano Membrane	5	4	28.3	38	751 052 294
Corpum A-Type Energized Adaptive Nano Membrane	5	4	28.3	38	752 390 745
Brokara’s Modified Energized Adaptive Nano Membrane	6	4	24.4	33	861 153 844
Tuvan’s Modified Energized Adaptive Nano Membrane	6	4	26.4	36	886 549 596
Raysere’s Modified Energized Adaptive Nano Membrane	6	4	26.4	36	1 161 748 215
Setele’s Modified Energized Adaptive Nano Membrane	6	4	28.3	38	1 395 125 182
Ahremen’s Modified Energized Adaptive Nano Membrane	6	4	28.3	38	1 536 937 438
Vizan’s Modified Energized Adaptive Nano Membrane	6	4	28.3	38	1 714 661 969
Cormack’s Modified Energized Adaptive Nano Membrane	6	4	30.3	40	10 170 313 575
Chelm’s Modified Energized Adaptive Nano Membrane	6	4	30.3	40	10 753 412 625
Draclira’s Modified Energized Adaptive Nano Membrane	6	4	30.3	40	11 027 470 045

Table S3: Energized Adaptive Nano Membrane module class data. Functional characteristics for each variant of the module class and average daily price for the item in the Forge region during 2015.

Module Name	MetaGroup	Rarity	Damage	CPU	Price (ISK)
Ballistic Control System I	1	1	15.03	35	65 969
Cross-linked Bolt Array I	1	3	16.59	37	346 039
Ballistic Control System II	2	1	21.55	40	816 551
Muon Coil Bolt Array I	1	3	18.16	39	816 603
Multiphasic Bolt Array I	1	3	19.74	40	2 139 205
‘Pandemonium’ Ballistic Enhancement	1	3	21.33	42	11 818 778
Ballistic ’Purge’ Targeting System I	3	4	18.25	30	26 122 686
‘Full Duplex’ Ballistic Targeting System	3	4	19.35	30	36 102 807
Domination Ballistic Control System	4	3	21.55	28	50 382 157
Republic Fleet Ballistic Control System	4	4	21.55	28	55 074 388
Caldari Navy Ballistic Control System	4	2	24.31	24	92 894 448
Dread Guristas Ballistic Control System	4	3	24.31	24	94 448 681
Khanid Navy Ballistic Control System	4	4	24.31	24	127 593 395
Mizuro’s Modified Ballistic Control System	6	4	22.1	31	435 538 765
Hakim’s Modified Ballistic Control System	6	4	22.66	34	444 063 110
Gotan’s Modified Ballistic Control System	6	4	23.21	36	561 835 333
Tobias’ Modified Ballistic Control System	6	4	23.76	39	945 333 333
Kaikka’s Modified Ballistic Control System	6	4	25	26	1 137 267 911
Thon’s Modified Ballistic Control System	6	4	25.69	29	1 900 513 954
Vepas’ Modified Ballistic Control System	6	4	26.38	31	4 023 867 248
Estamel’s Modified Ballistic Control System	6	4	27.08	34	10 021 052 707

Table S4: Ballistic Control System module class data. Functional characteristics for each variant of the module class and average daily price for the item in the Forge region during 2015.

Module Name	MetaGroup	Rarity	Damage	CPU	Price (ISK)
Insulated Stabilizer Array I	1	2	16.59	31	24 279
Linear Flux Stabilizer I	1	2	18.16	32	41 255
Magnetic Field Stabilizer I	1	1	15.03	30	73 102
Gauss Field Balancer I	1	2	19.74	33	74 662
Magnetic Vortex Stabilizer I	1	2	21.33	34	281 992
Magnetic Field Stabilizer II	2	1	21.55	30	838 337
'Capitalist' Magnetic Field Stabilizer I	3	4	19.9	27	19 075 706
'Monopoly' Magnetic Field Stabilizer I	3	4	21.55	24	53 218 239
Shadow Serpentis Magnetic Field Stabilizer	4	3	24.31	20	97 963 762
Federation Navy Magnetic Field Stabilizer	4	2	24.31	20	98 641 633
Brynn's Modified Magnetic Field Stabilizer	6	4	25.14	22	1 268 159 266
Tuvan's Modified Magnetic Field Stabilizer	6	4	25.69	24	2 201 000 233
Setele's Modified Magnetic Field Stabilizer	6	4	26.38	26	2 870 222 589
Cormack's Modified Magnetic Field Stabilizer	6	4	27.08	28	5 578 191 374

Table S5: Magnetic Field module class Stabilizer data. Functional characteristics for each variant of the module class and average daily price for the item in the Forge region during 2015.

Module Name	MetaGroup	Rarity	Damage	CPU	Price (ISK)
Thermal Exhaust System I	1	2	16.59	31	3 159
C4S Coiled Circuit Thermal Radiator	1	2	18.16	32	3 599
‘Skadi’ Coolant System I	1	2	19.74	33	5 664
Extruded Heat Sink I	1	2	21.33	34	32 182
Heat Sink I	1	2	15.03	30	42 268
Heat Sink II	2	1	21.55	30	676 955
‘Boreas’ Coolant System	1	4	13.42	26	4 435 363
C3S Convection Thermal Radiator	1	4	12.36	25	6 316 268
Heat Exhaust System	1	4	11.3	24	8 256 238
Stamped Heat Sink	1	4	14.49	28	9 021 492
Basic Heat Sink	1	4	10.25	20	11 597 890
‘Mangonel’ Heat Sink I	3	4	18.81	24	16 998 848
‘Trebuchet’ Heat Sink I	3	4	21	24	50 420 190
Ammatar Navy Heat Sink	4	4	24.31	20	84 398 523
Dark Blood Heat Sink	4	3	24.31	20	86 525 841
Imperial Navy Heat Sink	4	2	24.31	20	87 888 609
True Sansha Heat Sink	4	3	24.31	20	88 355 172
Tairei’s Modified Heat Sink	6	4	25	22	1 018 269 352
Brokara’s Modified Heat Sink	6	4	25	22	1 184 807 004
Raysere’s Modified Heat Sink	6	4	25.69	24	1 184 999 953
Selynne’s Modified Heat Sink	6	4	25.69	24	1 262 374 859
Ahremen’s Modified Heat Sink	6	4	26.38	26	2 132 015 979
Vizan’s Modified Heat Sink	6	4	26.38	26	2 227 597 757
Chelm’s Modified Heat Sink	6	4	27.08	28	4 039 947 018
Draclira’s Modified Heat Sink	6	4	27.08	28	4 130 799 959

Table S6: Heat Sink module class data. Functional characteristics for each variant of the module class and average daily price for the item in the Forge region during 2015.

Module Name	MetaGroup	Rarity	Damage	CPU	Price (ISK)
F-M3 Munition Inertial Suspensor	1	2	18.16	32	6 365
Cross-Lateral Gyrostabilizer I	1	2	16.59	31	7 220
Pneumatic Stabilization Actuator I	1	2	19.74	33	14 788
Counterbalanced Weapon Mounts I	1	2	21.33	34	44 785
Gyrostabilizer I	1	2	15.03	30	48 494
Gyrostabilizer II	2	1	21.55	30	677 997
Hydraulic Stabilization Actuator	1	4	13.42	26	4 381 223
Basic Gyrostabilizer	1	4	10.25	20	6 750 097
Lateral Gyrostabilizer	1	4	11.3	24	8 040 376
F-M2 Weapon Inertial Suspensor	1	4	12.36	25	8 278 197
Stabilized Weapon Mounts	1	4	14.49	28	11 356 878
'Kindred' Stabilization Actuator I	3	4	18.25	26	15 596 212
Republic Fleet Gyrostabilizer	4	2	24.31	20	64 807 737
Domination Gyrostabilizer	4	3	24.31	20	66 262 938
Mizuro's Modified Gyrostabilizer	6	4	25	22	939 347 718
Hakim's Modified Gyrostabilizer	6	4	25.69	24	1 828 302 841
Gotan's Modified Gyrostabilizer	6	4	26.38	26	3 541 756 371
Tobias' Modified Gyrostabilizer	6	4	27.08	28	6 551 400 700

Table S7: Gyrostabilizer module class data. Functional characteristics for each variant of the module class and average daily price for the item in the Forge region during 2015.

Module Name	MG	Rarity	Shield	CPU	Price (ISK)
Ionic Field Accelerator I	1	3	30	48	186 197
Shield Boost Amplifier I	1	2	30	50	217 983
5a Prototype Shield Support I	1	3	30	45	704 315
Shield Boost Amplifier II	2	2	36	55	706 831
'Stalwart' Particle Field Magnifier	1	3	30	43	2 032 232
'Copasetic' Particle Field Acceleration	1	3	30	40	11 663 931
'Glycerine' Shield Boost Amplifier	3	4	30	43	30 411 248
Domination Shield Boost Amplifier	4	3	33	40	39 816 118
Dread Guristas Shield Boost Amplifier	4	3	38	55	52 160 784
Gist C-Type Shield Boost Amplifier	5	4	36	54	61 451 498
Gist B-Type Shield Boost Amplifier	5	4	38	58	61 813 012
Pith C-Type Shield Boost Amplifier	5	3	39	61	73 115 355
Republic Fleet Shield Boost Amplifier	4	4	33	40	82 605 870
Pith B-Type Shield Boost Amplifier	5	3	41	66	86 058 500
Caldari Navy Shield Boost Amplifier	4	3	38	55	87 244 637
Gist A-Type Shield Boost Amplifier	5	4	39	63	87 435 510
Gist X-Type Shield Boost Amplifier	5	3	43	72	104 082 553
Pith A-Type Shield Boost Amplifier	5	3	43	72	119 944 957
Pith X-Type Shield Boost Amplifier	5	3	45	77	139 590 611
Kaikka's Modified Shield Boost Amplifier	6	4	39	61	315 686 510
Thon's Modified Shield Boost Amplifier	6	4	41	66	324 179 162
Vepas' Modified Shield Boost Amplifier	6	4	43	72	493 121 425
Tobias' Modified Shield Boost Amplifier	6	4	39	63	493 947 889
Hakim's Modified Shield Boost Amplifier	6	4	36	54	591 598 024
Estamel's Modified Shield Boost Amplifier	6	4	45	77	1 027 291 916

Table S8: Shield Boost Amplifier module class data. Functional characteristics for each variant of the module class and average daily price for the item in the Forge region during 2015.

Module Name	MG	Rarity	Shield	CPU	Capacitor	Price (ISK)
Adaptive Invulnerability Field I	1	1	25	40	40	116 501
Limited Adaptive Invulnerability Field I	1	1	25	34	40	122 351
Adaptive Invulnerability Field II	2	1	30	44	32	1 653 130
Domination Adaptive Invulnerability Field	4	3	25	34	16	62 737 981
‘Posse’ Adaptive Invulnerability Field	3	4	27.5	34	40	64 293 250
Dread Guristas Adaptive Invulnerability Field	4	3	37.5	27	40	197 788 502
Gistum C-Type Adaptive Invulnerability Field	5	3	37.5	27	32	210 313 698
Pithum C-Type Adaptive Invulnerability Field	5	2	40.63	29	40	258 065 588
Gistum B-Type Adaptive Invulnerability Field	5	3	40.63	30	34	313 307 707
Caldari Navy Adaptive Invulnerability Field	4	4	37.5	27	40	379 591 119
Pithum B-Type Adaptive Invulnerability Field	5	3	43.75	32	40	477 112 531
Gistum A-Type Adaptive Invulnerability Field	5	4	43.75	33	36	640 494 487
Pithum A-Type Adaptive Invulnerability Field	5	3	46.88	35	40	822 615 600
Kaikka’s Modified Adaptive Invulnerability Field	6	4	40.63	29	40	901 205 414
Thon’s Modified Adaptive Invulnerability Field	6	4	43.75	32	40	1 465 849 881
Vepas’s Modified Adaptive Invulnerability Field	6	4	46.88	35	40	3 867 136 734
Estamel’s Modified Adaptive Invulnerability Field	6	4	50	37	40	25 750 650 000

Table S9: Adaptive Invulnerability Field module class data. Functional characteristics for each variant of the module class and average daily price for the item in the Forge region during 2015.

Module Name	MG	Rarity	Armor	CPU	Capacitor	Price (ISK)
Armor Explosive Hardener I	1	2	50	33	30	141 193
Upgraded Armor Explosive Hardener I	1	2	50	31	29	307 192
Limited Armor Explosive Hardener I	1	2	50	30	27	308 124
Prototype Armor Explosive Hardener I	1	2	50	26	24	308 815
Experimental Armor Explosive Hardener I	1	2	50	28	26	312 887
Armor Explosive Hardener II	2	2	55	36	30	1 598 938
Domination Armor Explosive Hardener	4	4	50	33	10	3 838 685
Republic Fleet Armor Explosive Hardener	4	4	50	33	10	12 363 898
Shadow Serpentis Armor Explosive Hardener	4	3	55	16	30	14 572 755
Dark Blood Armor Explosive Hardener	4	3	55	16	30	15 097 140
True Sansha Armor Explosive Hardener	4	3	55	16	30	16 830 406
Imperial Navy Armor Explosive Hardener	4	4	55	16	30	32 165 149
Federation Navy Armor Explosive Hardener	4	4	55	16	30	33 679 231
Core C-Type Armor Explosive Hardener	5	4	57.3	18	34	44 606 520
Ammatar Navy Armor Explosive Hardener	4	4	55	16	30	44 714 285
Centus C-Type Armor Explosive Hardener	5	4	57.3	18	34	44 737 312
Corpus C-Type Armor Explosive Hardener	5	4	57.3	18	34	51 376 745
Centus B-Type Armor Explosive Hardener	5	4	59.5	20	38	60 598 370
Core B-Type Armor Explosive Hardener	5	4	59.5	20	38	61 032 295
Corpus B-Type Armor Explosive Hardener	5	4	59.5	20	38	63 400 277
Core A-Type Armor Explosive Hardener	5	4	61.8	21	42	87 348 641
Centus A-Type Armor Explosive Hardener	5	4	61.8	21	42	91 895 146
Corpus A-Type Armor Explosive Hardener	5	4	61.8	21	42	94 763 710
Centus X-Type Armor Explosive Hardener	5	3	64	23	46	109 727 564
Corpus X-Type Armor Explosive Hardener	5	3	64	23	46	111 312 664
Core X-Type Armor Explosive Hardener	5	3	64	23	46	111 640 276
Khanid Navy Armor Explosive Hardener	4	4	55	16	30	174 012 092
Raysere’s Modified Armor Explosive Hardener	6	4	59.5	20	38	225 913 500
Tuvan’s Modified Armor Explosive Hardener	6	4	59.5	20	38	248 986 411
Brokara’s Modified Armor Explosive Hardener	6	4	57.3	18	34	296 667 222
Brynn’s Modified Armor Explosive Hardener	6	4	57.3	18	34	353 085 972
Tairei’s Modified Armor Explosive Hardener	6	4	57.3	18	34	365 872 139
Ahremen’s Modified Armor Explosive Hardener	6	4	61.8	21	42	435 062 375
Selynne’s Modified Armor Explosive Hardener	6	4	59.5	20	38	491 202 707
Setele’s Modified Armor Explosive Hardener	6	4	61.8	21	42	538 520 989
Cormack’s Modified Armor Explosive Hardener	6	4	64	23	46	893 066 737
Chelm’s Modified Armor Explosive Hardener	6	4	64	23	46	1 218 899 905
Vizan’s Modified Armor Explosive Hardener	6	4	61.8	21	42	1 435 216 243
Draclira’s Modified Armor Explosive Hardener	6	4	64	23	46	1 450 000 000

Table S10: Armor Explosive Hardener module class data. Functional characteristics for each variant of the module class and average daily price for the item in the Forge region during 2015.

Module Name	MG	Rarity	Armor	CPU	Power	Capacitor	Price (ISK)
Large Inefficient Armor Repair Unit	1	2	759	48	1800	400	29 525
Large Vestment Reconstructer I	1	2	828	46	1800	400	29 836
Large I-a Polarized Armor Regenerator	1	2	725	47	1800	400	30 255
Large Automated Carapace Restoration	1	2	794	49	1800	400	30 566
Large Armor Repairer I	1	3	690	50	1800	400	113 533
Large Armor Repairer II	2	2	920	55	2070	400	1 333 400
Shadow Serpentis Large Armor Repairer	4	3	920	46	1800	400	13 162 616
Domination Large Armor Repairer	4	4	828	46	1800	360	13 650 309
True Sansha Large Armor Repairer	4	3	1035	46	1800	450	42 040 990
Dark Blood Large Armor Repairer	4	4	1035	46	1800	450	42 474 197
Ammatar Navy Large Armor Repairer	4	4	1035	46	1800	450	47 147 176
Federation Navy Large Armor Repairer	4	4	920	46	1800	400	64 340 301
Core C-Type Large Armor Repairer	5	4	1012	46	1980	400	74 850 997
Republic Fleet Large Armor Repairer	4	4	828	46	1800	360	81 143 325
Khanid Navy Large Armor Repairer	4	4	1035	46	1800	450	83 371 999
Centus C-Type Large Armor Repairer	5	4	1139	46	1980	450	101 346 923
Core B-Type Large Armor Repairer	5	4	1104	46	2160	400	101 600 800
‘Hauberk’ Large Armor Repairer I	3	4	920	40	1620	400	109 901 496
Corpus C-Type Large Armor Repairer	5	4	1139	46	1980	450	112 471 318
‘Protest’ Large Armor Repairer I	3	4	1035	40	1620	400	134 913 901
Imperial Navy Large Armor Repairer	4	4	1035	46	1800	450	152 024 142
Centus B-Type Large Armor Repairer	5	4	1242	46	2160	450	158 502 884
Corpus B-Type Large Armor Repairer	5	4	1242	46	2160	450	166 348 573
Core A-Type Large Armor Repairer	5	4	1196	46	2340	400	167 398 342
Core X-Type Large Armor Repairer	5	3	1288	46	2520	400	213 278 900
Brynn’s Modified Large Armor Repairer	6	4	1012	46	1980	400	271 449 390
Tuvan’s Modified Large Armor Repairer	6	4	1104	46	2160	400	274 414 950
Brokara’s Modified Large Armor Repairer	6	4	1139	46	1980	450	296 110 254
Corpus A-Type Large Armor Repairer	5	4	1346	46	2340	450	297 691 628
Centus A-Type Large Armor Repairer	5	4	1346	46	2340	450	302 189 114
Centus X-Type Large Armor Repairer	5	3	1449	46	2520	450	345 698 565
Corpus X-Type Large Armor Repairer	5	3	1449	46	2520	450	355 303 674
Tairei’s Modified Large Armor Repairer	6	4	1139	46	1980	450	370 595 669
Mizuro’s Modified Large Armor Repairer	6	4	911	46	1890	360	380 154 887
Setele’s Modified Large Armor Repairer	6	4	1196	46	2340	400	386 889 714
Raysere’s Modified Large Armor Repairer	6	4	1242	46	2160	450	487 571 397
Selynne’s Modified Large Armor Repairer	6	4	1242	46	2160	450	622 300 359
Ahremen’s Modified Large Armor Repairer	6	4	1346	46	2340	450	638 889 111
Vizan’s Modified Large Armor Repairer	6	4	1346	46	2340	450	642 062 625
Gotan’s Modified Large Armor Repairer	6	4	1076	46	2070	360	763 314 245
Cormack’s Modified Large Armor Repairer	6	4	1288	46	2520	400	770 375 101
Chelm’s Modified Large Armor Repairer	6	4	1449	46	2520	450	1 168 786 215
Draclira’s Modified Large Armor Repairer	6	4	1449	46	2520	450	1 199 953 331

Table S11: Large Armor Repair Unit module class data. Functional characteristics for each variant of the module class and average daily price for the item in the Forge region during 2015.

Module Name	MG	Rarity	Shield	CPU	Power	Capacitor	Price (ISK)
X-Large Clarity Ward Booster I	1	2	119.2	160	500	80	120 451
X-Large Neutron Saturation Injector I	1	2	113.8	180	500	80	121 510
X-Large Converse Deflection Catalyzer	1	2	108.8	190	500	80	123 656
X-Large C5-L Emergency Shield Overload I	1	2	124.2	170	500	80	124 266
X-Large Shield Booster I	1	3	103.6	200	500	80	390 376
X-Large Shield Booster II	2	3	138	230	550	80	1 632 874
Dread Guristas X-Large Shield Booster	4	3	157.5	170	500	100	11 472 249
Domination X-Large Shield Booster	4	4	138	170	500	80	23 029 241
X-Large 'Locomotive' Shield Booster	3	4	110.4	170	450	80	28 008 742
Pith C-Type X-Large Shield Booster	5	3	181.5	187	500	100	30 593 729
Pith B-Type X-Large Shield Booster	5	3	198	204	500	100	35 852 899
Pith A-Type X-Large Shield Booster	5	3	214.5	221	500	100	59 593 766
Pith X-Type X-Large Shield Booster	5	3	231	238	500	100	74 903 496
Republic Fleet X-Large Shield Booster	4	4	138	170	500	80	113 472 639
Caldari Navy X-Large Shield Booster	4	4	157.5	170	500	100	123 073 376
Gist C-Type X-Large Shield Booster	5	4	144.5	170	500	44.75	384 442 387
Kaikka's Modified X-Large Shield Booster	6	4	181.5	187	500	100	397 064 175
Gist B-Type X-Large Shield Booster	5	4	158.5	170	500	46.75	408 621 296
Thon's Modified X-Large Shield Booster	6	4	198	204	500	100	451 418 841
Gist A-Type X-Large Shield Booster	5	4	170.5	170	500	49	487 802 227
Vepas' Modified X-Large Shield Booster	6	4	214.5	221	500	100	566 227 640
Gist X-Type X-Large Shield Booster	5	3	184.75	170	500	51	569 580 609
Hakim's Modified X-Large Shield Booster	6	4	158.5	170	500	46.75	876 269 231
Estamel's Modified X-Large Shield Booster	6	4	231	238	500	100	1 264 654 857
Tobias' Modified X-Large Shield Booster	6	4	184.75	170	500	51	3 550 250 000

Table S12: X-Large Shield Booster module class data. Functional characteristics for each variant of the module class and average daily price for the item in the Forge region during 2015.

Module Name	MG	Rarity	Shield	CPU	Power	Capacitor	Price (ISK)
Large Neutron Saturation Injector I	1	2	57	90	150	40	42 369
Large C5-L Emergency Shield Overload I	1	2	62	85	150	40	42 565
Large Clarity Ward Booster I	1	2	59.5	80	150	40	42 787
Large Converse Deflection Catalyzer	1	2	54.25	95	150	40	43 212
Large Shield Booster I	1	3	51.75	100	150	40	124 112
Large Shield Booster II	2	2	69	115	165	40	878 442
Dread Guristas Large Shield Booster	4	3	78.75	85	150	50	4 712 231
Domination Large Shield Booster	4	4	69.06	85	150	40	8 262 317
Large ‘Outlaw’ Shield Booster	3	4	55.25	85	135	40	14 499 525
Pith C-Type Large Shield Booster	5	3	90.63	94	150	50	40 383 848
Republic Fleet Large Shield Booster	4	4	69.06	85	150	40	40 776 563
Pith B-Type Large Shield Booster	5	3	99.06	102	150	50	57 670 645
Caldari Navy Large Shield Booster	4	4	78.75	85	150	50	63 729 681
Pith A-Type Large Shield Booster	5	3	107.19	111	150	50	101 266 063
Pith X-Type Large Shield Booster	5	3	115.63	119	150	50	110 258 929
Gist C-Type Large Shield Booster	5	4	74.06	85	150	28.13	146 988 292
Gist B-Type Large Shield Booster	5	4	79.06	85	150	29.38	156 148 159
Kaikka’s Modified Large Shield Booster	6	4	90.63	94	150	50	204 053 591
Gist A-Type Large Shield Booster	5	4	84.38	85	150	30.63	207 636 485
Gist X-Type Large Shield Booster	5	3	92.5	85	150	31.88	219 231 057
Hakim’s Modified Large Shield Booster	6	4	79.06	85	150	29.38	327 659 630
Thon’s Modified Large Shield Booster	6	4	99.06	102	150	50	392 660 044
Vepas’ Modified Large Shield Booster	6	4	107.19	111	150	50	598 911 917
Tobias’ Modified Large Shield Booster	6	4	92.5	85	150	31.88	737 850 089
Estamel’s Modified Large Shield Booster	6	4	115.63	119	150	50	860 071 386

Table S13: Large Shield Booster module class data. Functional characteristics for each variant of the module class and average daily price for the item in the Forge region during 2015.

Module Name	MG	Rarity	Range	Strength	CPU	Power	Price (ISK)
‘Langour’ Drive Disruptor I	1	1	10000	52.5	20	1	16 425
Patterned Stasis Web I	1	2	10000	55	23	1	40 088
Stasis Webifier I	1	2	10000	50	25	1	43 107
X5 Prototype Engine Enervator	1	1	10000	57.5	21	1	273 307
Stasis Webifier II	2	1	10000	60	30	1	675 543
Fleeting Propulsion Inhibitor I	1	1	10000	60	22	1	2 056 796
Caldari Navy Stasis Webifier	4	3	12000	55	18	1	38 984 748
Dread Guristas Stasis Webifier	4	3	13000	50	18	1	40 075 978
Shadow Serpentis Stasis Webifier	4	3	13000	55	25	1	42 987 772
Khanid Navy Stasis Webifier	4	4	12000	60	25	1	64 508 034
Federation Navy Stasis Webifier	4	2	14000	60	25	1	67 283 514
Dark Blood Stasis Webifier	4	3	14000	55	18	1	67 328 108
Domination Stasis Webifier	4	3	15000	50	18	1	120 141 214
True Sansha Stasis Webifier	4	3	15000	55	25	1	163 459 991
Mizuro’s Modified Stasis Webifier	6	4	17000	55	18	1900	2 187 846 687
Hakim’s Modified Stasis Webifier	6	4	18000	60	25	2100	4 814 356 341
Gotan’s Modified Stasis Webifier	6	4	19000	57.5	18	1800	6 004 110 534
Tobias’ Modified Stasis Webifier	6	4	20000	60	18	2200	10 142 085 519

Table S14: Stasis Webifier module class data. Functional characteristics for each variant of the module class and average daily price for the item in the Forge region during 2015.

Module Name	MG	Rarity	Range	Strength	CPU	Power	Capacitor	Price (ISK)
Initiated Warp Disruptor I	1	2	20000	1	38	1	24	9 812
Fleeting Warp Disruptor I	1	2	20000	1	36	1	20	13 528
J5 Prototype Warp Disruptor I	1	1	20000	1	34	1	23	14 248
Warp Disruptor I	1	2	20000	1	40	1	25	63 522
Khanid Navy Warp Disruptor	4	4	24000	1	40	1	25	70 001
Faint Warp Disruptor I	1	2	20000	1	32	1	21	207 097
Warp Disruptor II	2	1	24000	1	44	1	25	1 067 043
Dread Guristas Warp Disruptor	4	3	24000	1	40	1	25	17 731 564
Process-Interruptive Warp Disruptor	3	4	22000	1	34	1	25	25 140 431
Caldari Navy Warp Disruptor	4	4	24000	1	40	1	25	27 505 144
Shadow Serpentis Warp Disruptor	4	3	26000	1	40	1	25	56 660 135
Dark Blood Warp Disruptor	4	3	28000	1	40	1	25	92 437 085
True Sansha Warp Disruptor	4	3	28000	1	40	1	25	93 696 427
Republic Fleet Warp Disruptor	4	2	30000	1	19	1	28	149 593 860
Domination Warp Disruptor	4	3	30000	1	19	1	28	167 672 697
Mizuro's Modified Warp Disruptor	6	4	25000	2	19	2500	50	905 092 825
Hakim's Modified Warp Disruptor	6	4	30000	2	19	3000	60	2 340 854 373
Gotan's Modified Warp Disruptor	6	4	35000	2	19	3500	70	6 609 279 185
Tobias' Modified Warp Disruptor	6	4	40000	2	19	4000	80	13 866 500 001

Table S15: Warp Disruptor module class data. Functional characteristics for each variant of the module class and average daily price for the item in the Forge region during 2015.

Module Name	MG	Rarity	Range	Strength	CPU	Power	Capacitor	Price (ISK)
Fleeting Progressive Warp Scrambler I	1	2	8250	2	29	1	4	24 685
Initiated Harmonic Warp Scrambler I	1	1	7875	2	26	1	5	25 831
Warp Scrambler I	1	1	7500	2	30	1	5	70 609
J5b Prototype Warp Scrambler I	1	1	8625	2	27	1	5	97 328
Warp Scrambler II	2	1	9000	2	36	1	5	1 211 082
Faint Epsilon Warp Scrambler I	1	1	9000	2	28	1	4	3 252 827
Caldari Navy Warp Scrambler	4	3	9750	2	22	1	5	29 757 553
'Delineative' Warp Scrambler	3	4	9000	2	18	1	5	40 671 079
Dread Guristas Warp Scrambler	4	3	10500	2	26	1	5	75 468 415
Dark Blood Warp Scrambler	4	3	10500	2	22	1	5	92 050 759
Khanid Navy Warp Scrambler	4	4	9750	3	26	1	5	106 572 813
Shadow Serpentis Warp Scrambler	4	3	10500	3	30	1	5	113 128 051
Republic Fleet Warp Scrambler	4	4	10500	3	26	1	6	142 966 296
Domination Warp Scrambler	4	3	11250	2	26	1	6	177 283 761
True Sansha Warp Scrambler	4	3	11250	3	30	1	5	198 444 978
Mizuro's Modified Warp Scrambler	6	4	12750	3	22	2500	15	1 139 527 071
Hakim's Modified Warp Scrambler	6	4	13500	3	22	2650	15	2 050 758 527
Gotan's Modified Warp Scrambler	6	4	14250	3	22	2800	15	3 143 419 901
Tobias' Modified Warp Scrambler	6	4	15000	3	22	3000	15	6 273 333 397

Table S16: Warp Scrambler module class data. Functional characteristics for each variant of the module class and average daily price for the item in the Forge region during 2015.

ID	Linear Model			Log-linear Functional			Log-Linear Social Categ.			Log-linear Social Discrete		
	R2	Adj R2	AIC	R2	Adj R2	AIC	R2	Adj R2	AIC	R2	Adj R2	AIC
1	0.342	0.19	814.6	0.845	0.809	64.4	0.964	0.947	43.6	0.974	0.963	37.9
2	0.308	0.264	1617.8	0.954	0.951	82.2	0.977	0.974	62.1	0.986	0.984	43.9
3	0.372	0.302	959.8	0.792	0.768	81.1	0.973	0.966	42.5	0.977	0.971	39.2
4	0.373	0.248	1089.1	0.748	0.697	104.5	0.982	0.976	42	0.982	0.976	42.7
5	0.539	0.49	1775.6	0.817	0.798	152.4	0.963	0.957	87.8	0.959	0.952	92
6	0.827	0.774	811.9	0.903	0.873	69.7	0.968	0.951	53.6	0.967	0.949	54.2
7	0.292	0.228	1033.4	0.692	0.664	91.5	0.92	0.904	61.8	0.919	0.903	62
8	0.96	0.944	838.1	0.86	0.806	85.5	0.887	0.816	85.3	0.911	0.855	80.8
9	0.498	0.398	1027.4	0.782	0.738	103.2	0.975	0.966	53.2	0.964	0.952	62.1
10	0.67	0.56	674.9	0.57	0.427	92	0.988	0.981	34.8	0.972	0.955	49.5
11	0.852	0.795	833.5	0.923	0.894	68.2	0.949	0.917	64.4	0.964	0.94	58.1
12	0.322	0.263	1644	0.853	0.841	114.7	0.946	0.938	79.8	0.976	0.973	47.6
13	0.252	0.23	1552.6	0.751	0.744	153.4	0.946	0.941	102.4	0.91	0.902	120.6
14	0.576	0.499	626.9	0.888	0.868	56.6	0.978	0.968	38	0.985	0.978	32.5
15	0.403	0.349	1109.3	0.67	0.64	123.6	0.918	0.902	92.7	0.902	0.882	97.2
16	0.344	0.256	814	0.674	0.63	89.9	0.922	0.898	68.2	0.917	0.892	69.2

Table S17: Regression results by module class. Summary table of the regression results across all 16 module classes included in our analysis.

3 Additional Figures

3.1 Times Series of Prices

Times series of the ln of daily average prices in the Forge region during 2015 for all 16 module classes in our analysis. Note that, unlike the price time series figure in the text, the colors in these plots do not correspond to the MetaGroup, but are merely a spectrum from low to high.

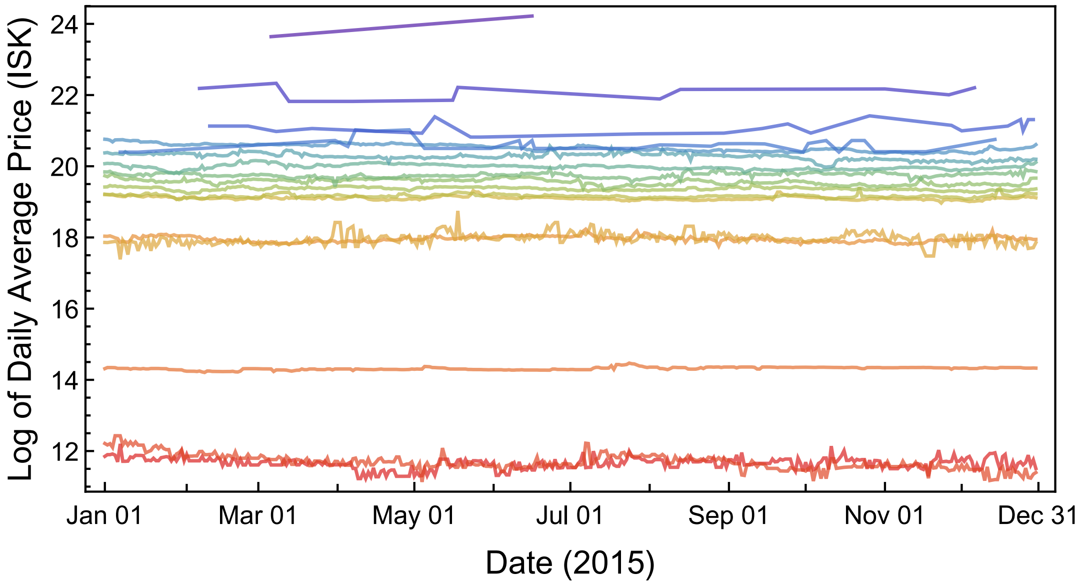

Figure S2: Price time series for the Adaptive Nano Plating module class.

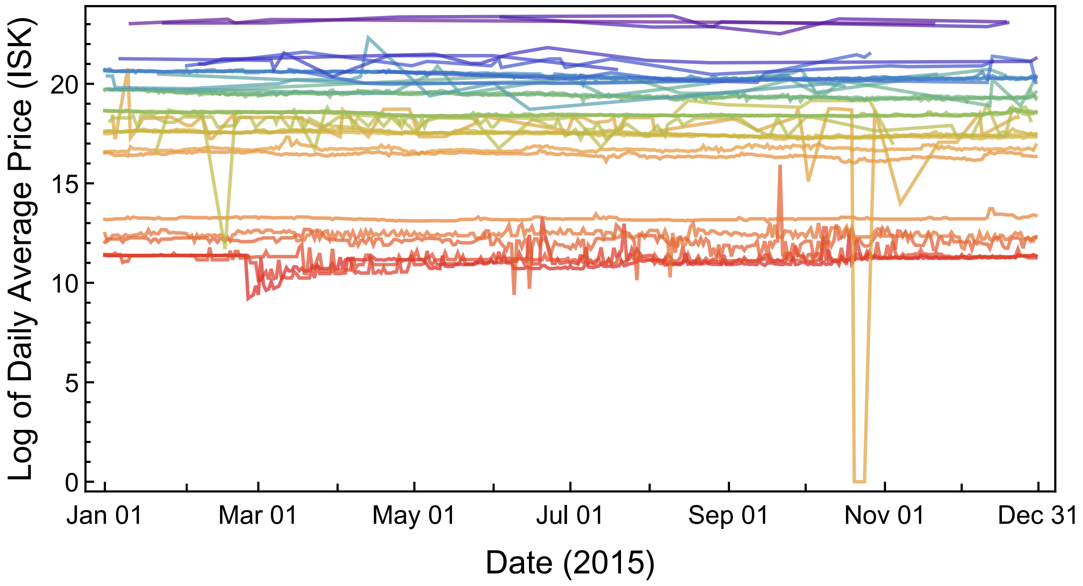

Figure S3: Price time series for the Energized Adaptive Nano Membrane module class.

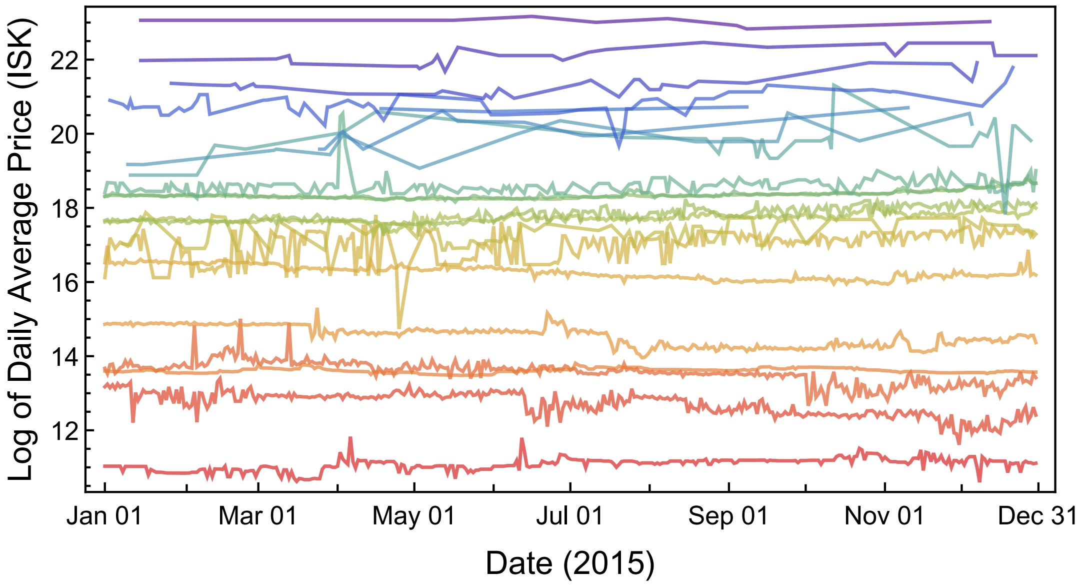

Figure S4: Price time series for the Ballistic Control System module class.

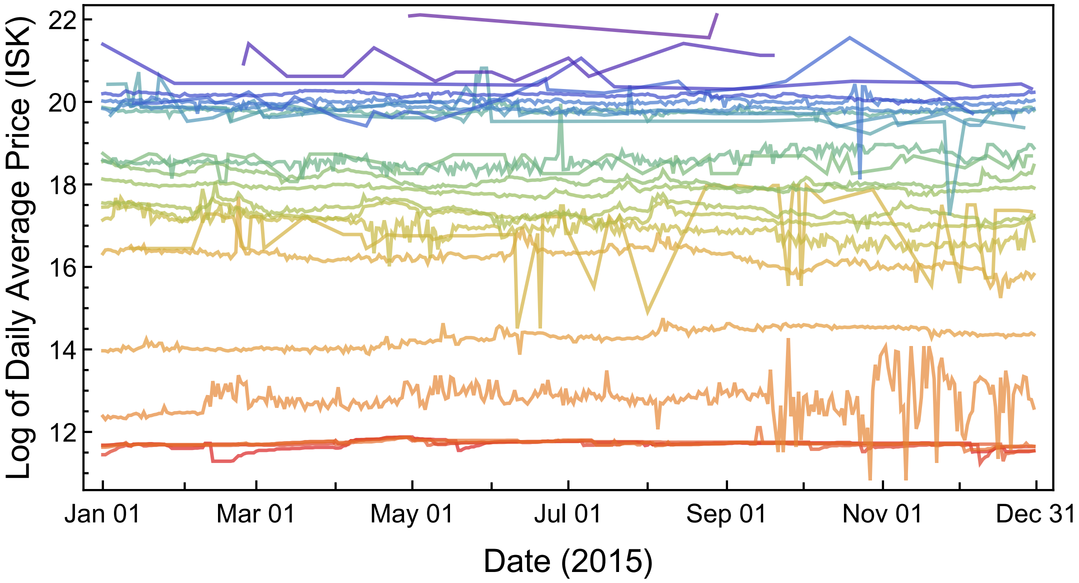

Figure S5: Price time series for the Magnetic Field Stabilizer module class.

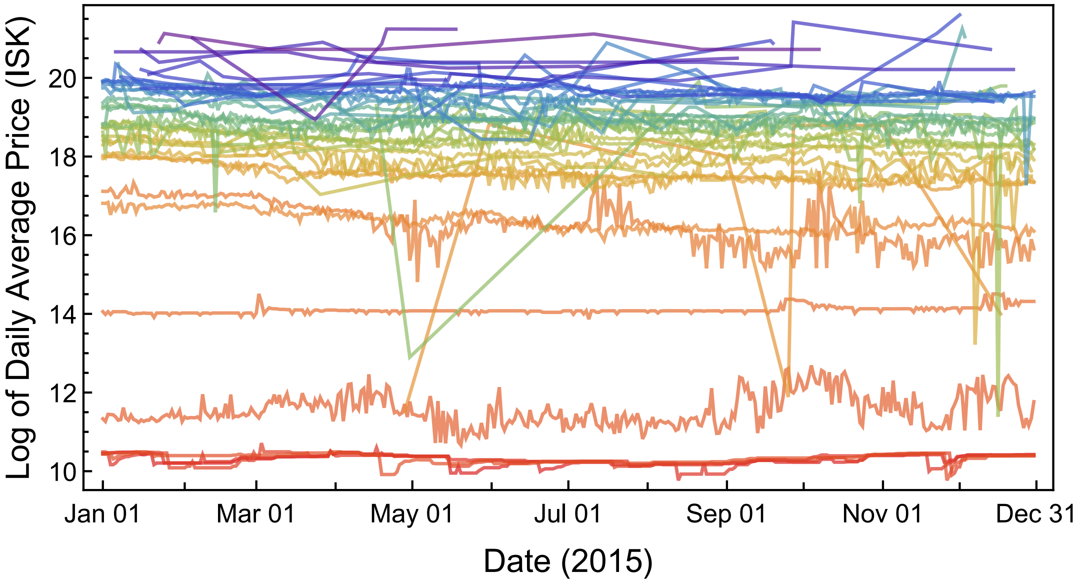

Figure S6: Price time series for the Heat Sink module class.

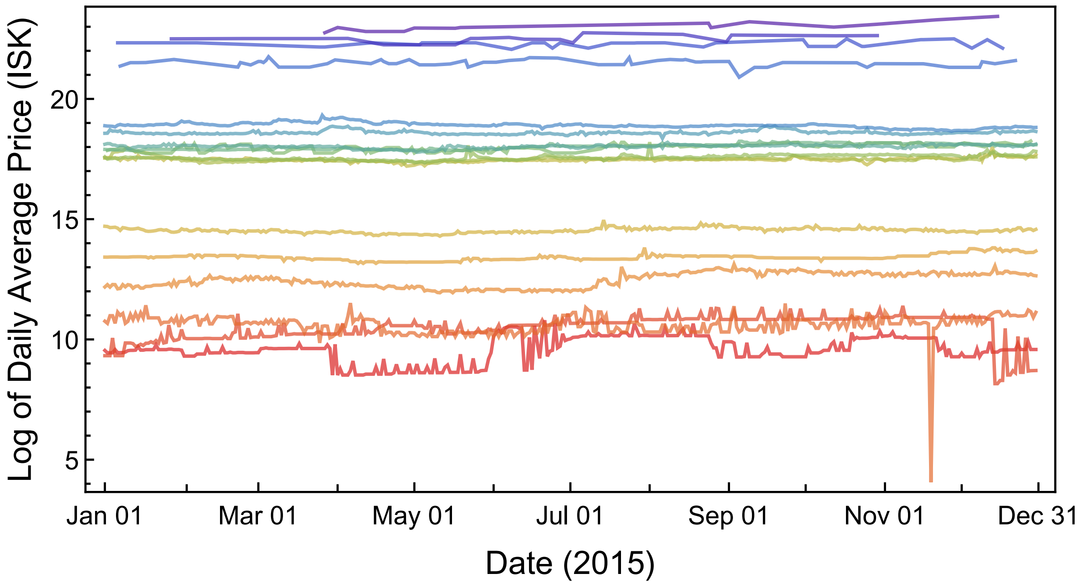

Figure S7: Price time series for the Gyrostabilizer module class.

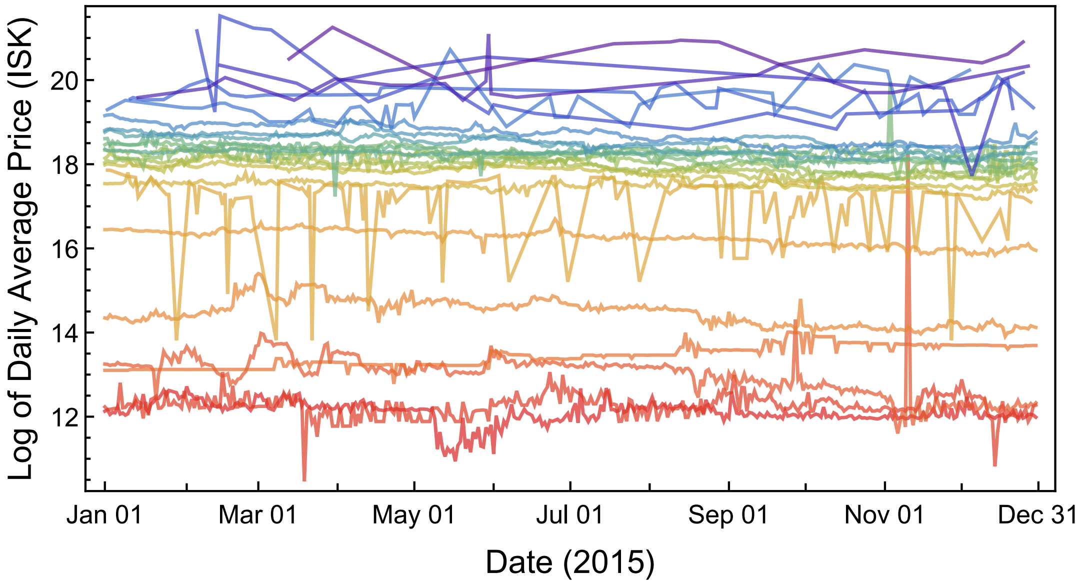

Figure S8: Price time series for the Shield Boost Amplifier module class.

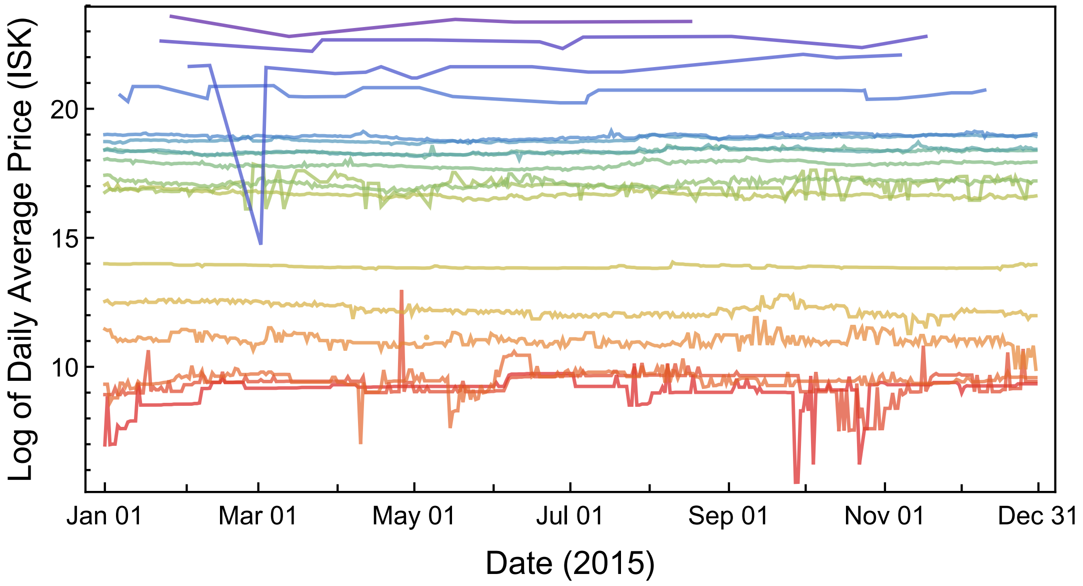

Figure S9: Price time series for the Adaptive Invulnerability Field module class.

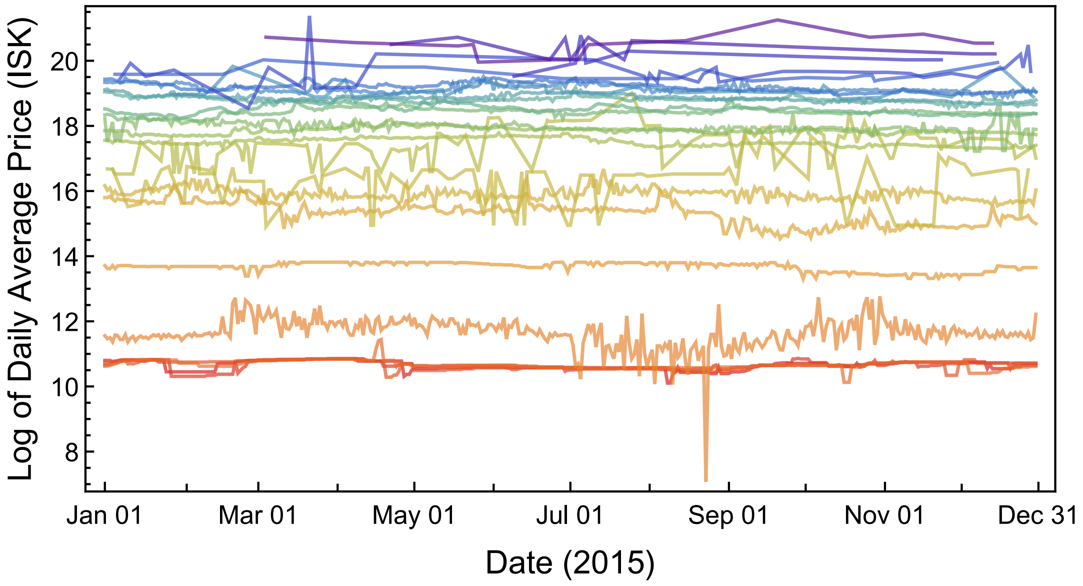

Figure S10: Price time series for the Armor Explosive Hardener module class.

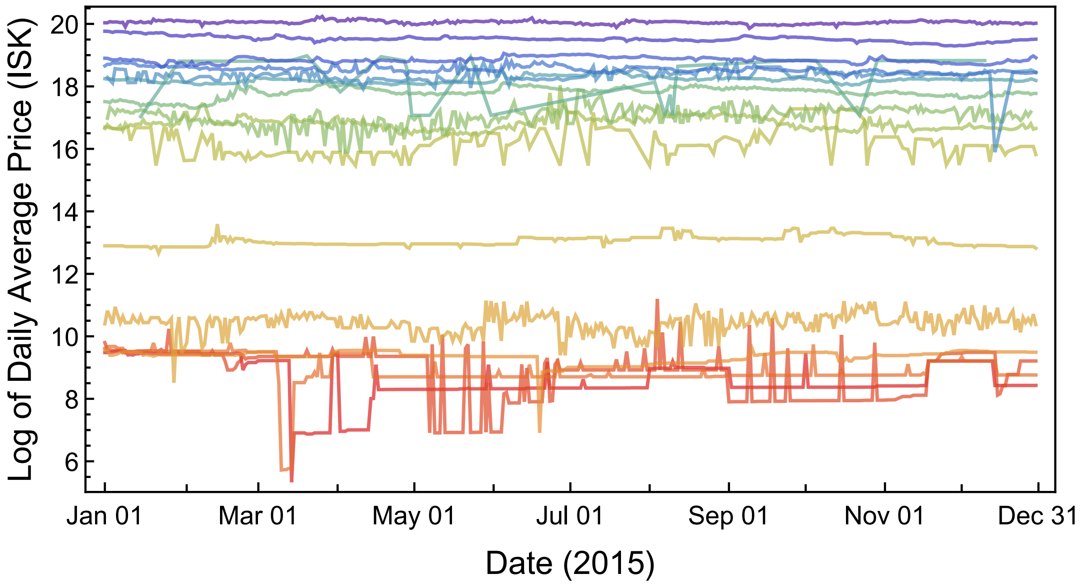

Figure S11: Price time series for the Large Armor Repair Unit module class.

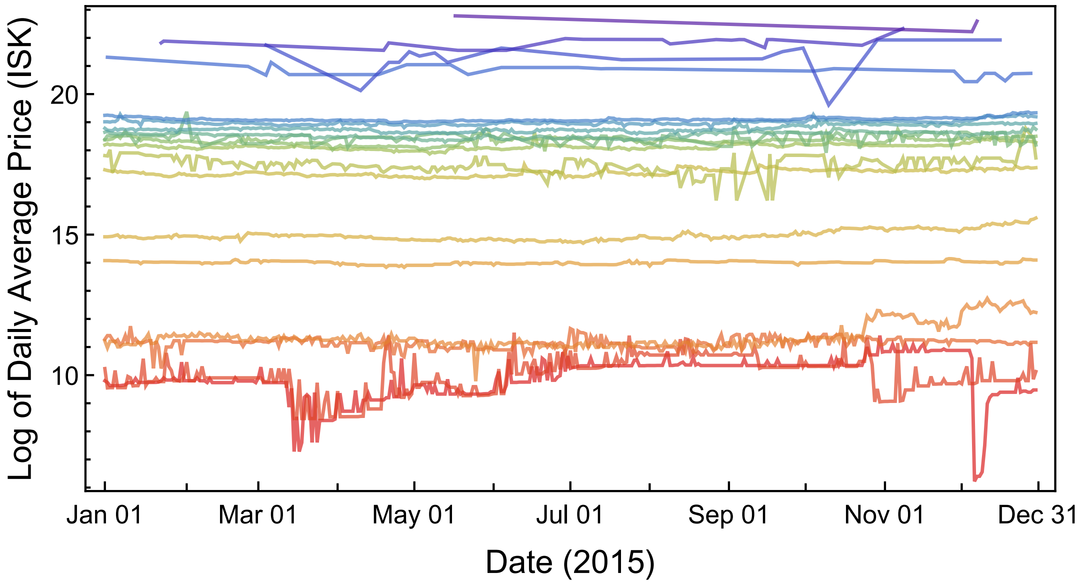

Figure S12: Price time series for the X-Large Shield Booster module class.

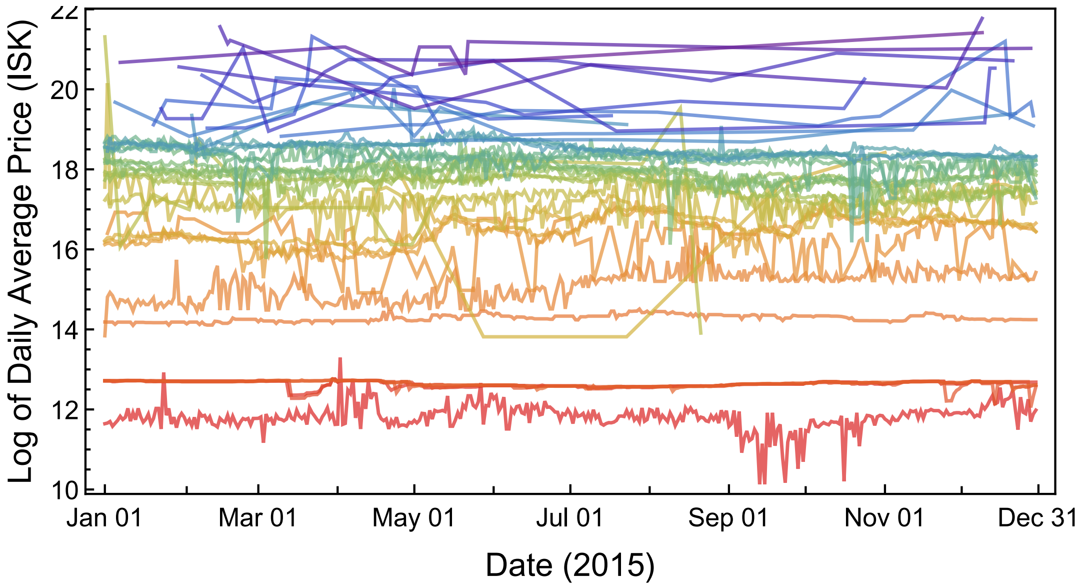

Figure S13: Price time series for the Large Shield Booster module class.

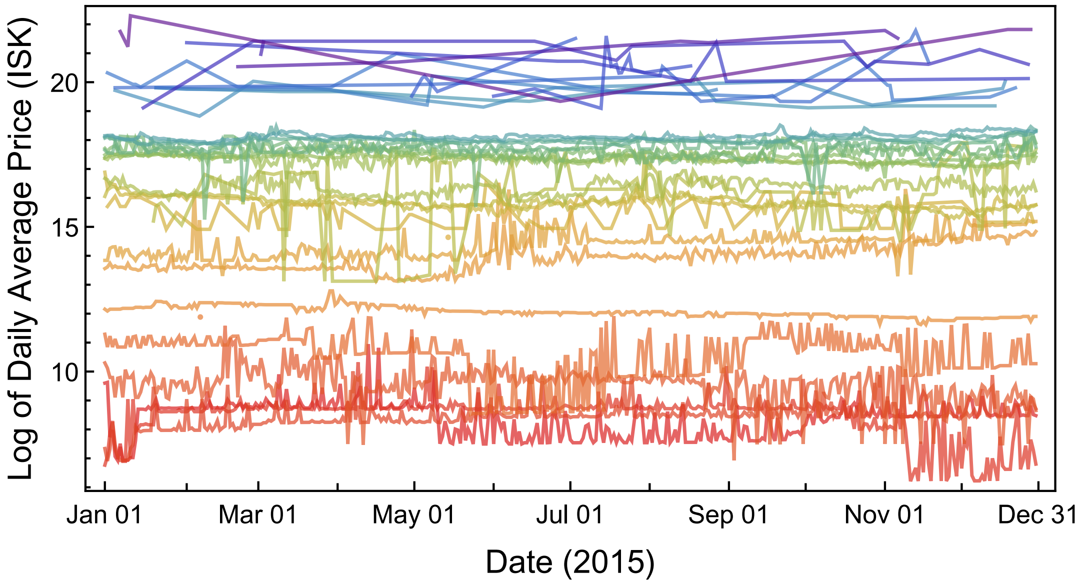

Figure S14: Price time series for the Medium Shield Booster module class.

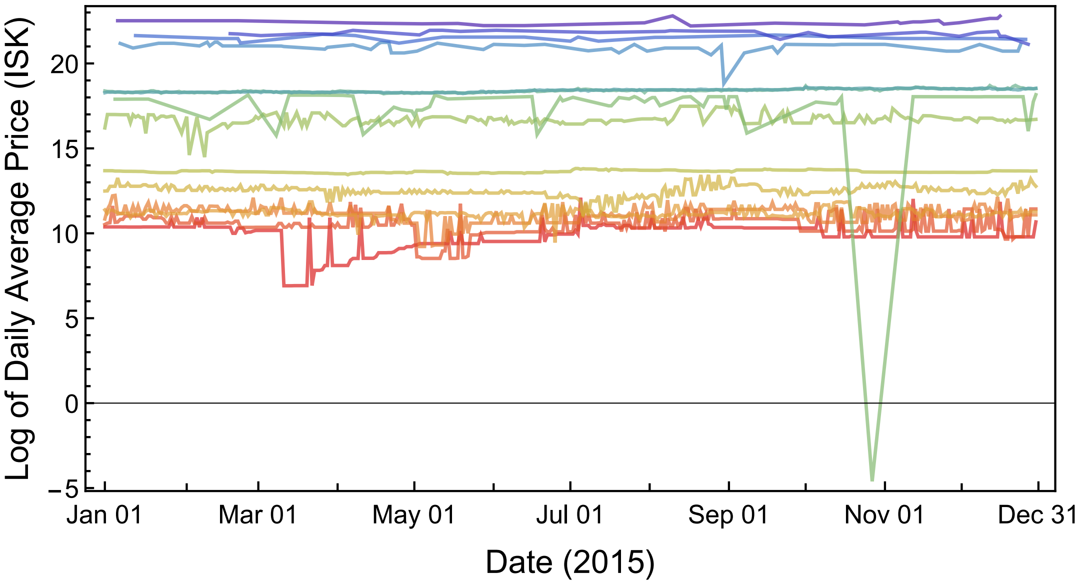

Figure S15: Price time series for the Stasis Webifier module class.

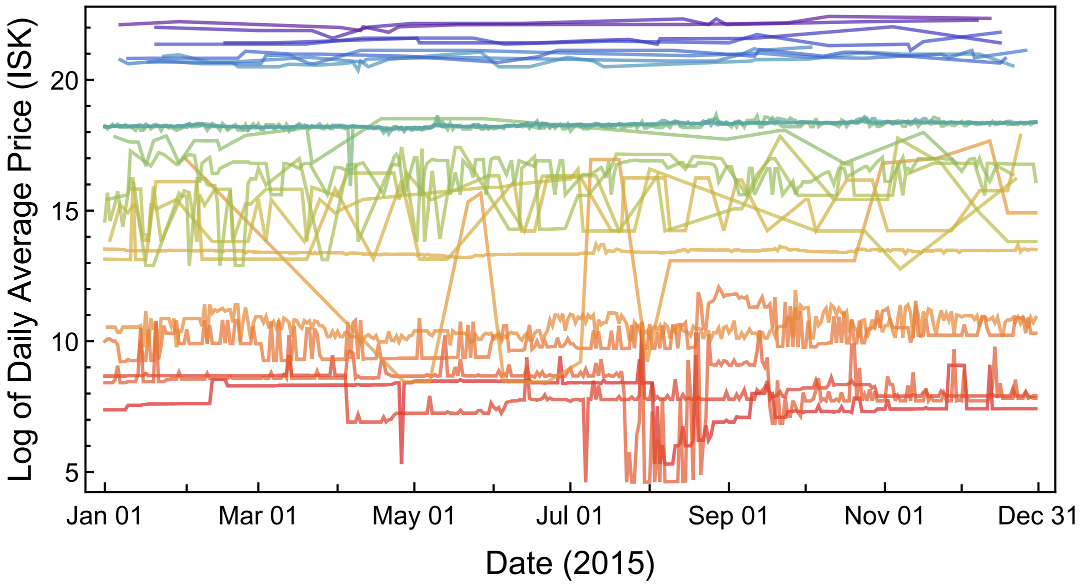

Figure S16: Price time series for the Warp Disruptor module class.

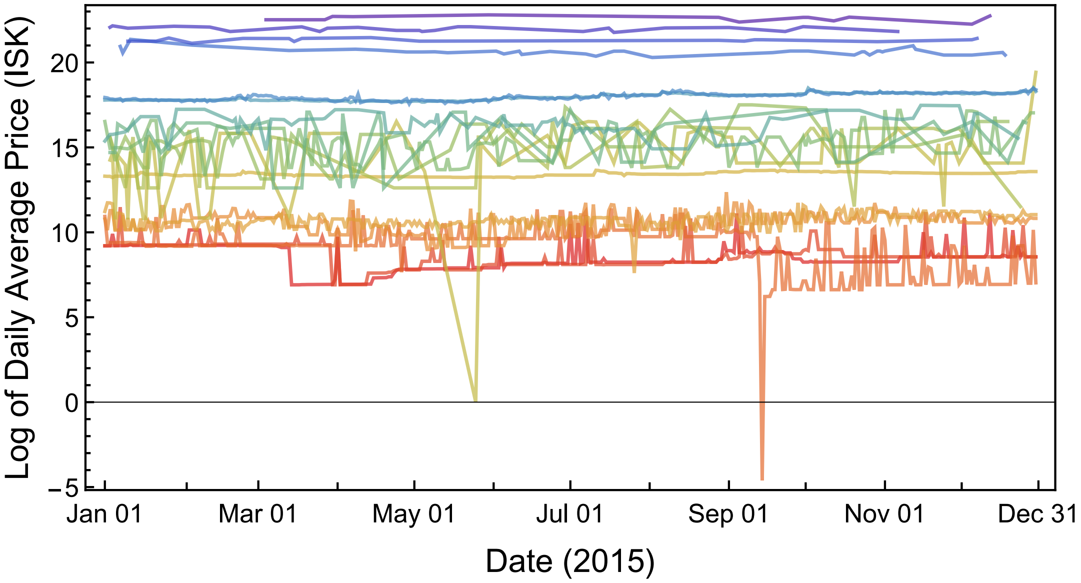

Figure S17: Price time series for the Warp Scrambler module class.

3.2 Goodness of fit

Plots of the log-linear functional and social-value models alongside the actual log prices to show the relative goodnesses of fit across all 16 modules.

Actual Ln Price   Functional Model Prediction   Social Value Model Prediction

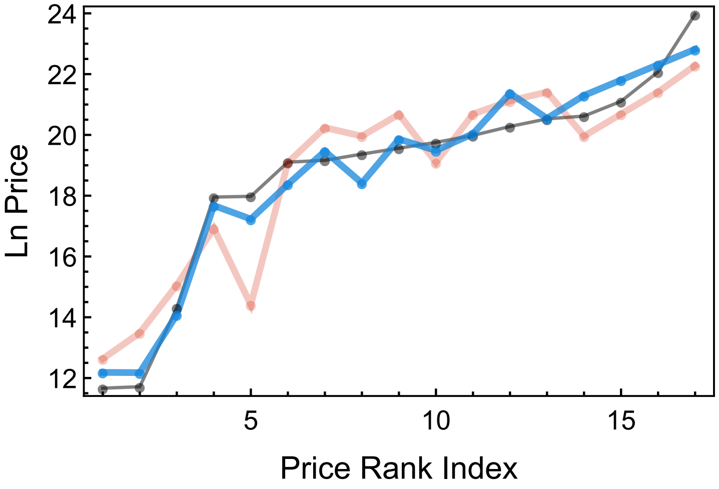

Figure S18: Real and predicted values for the Adaptive Nano Plating module class.

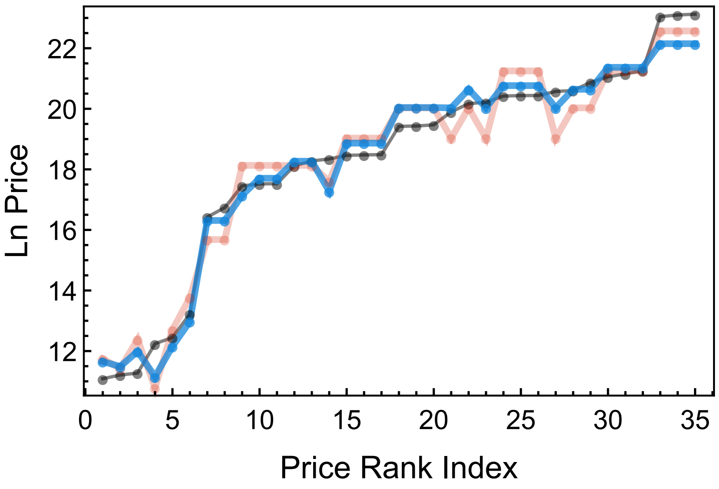

Figure S19: Real and predicted values for the Energized Adaptive Nano Membrane module class.

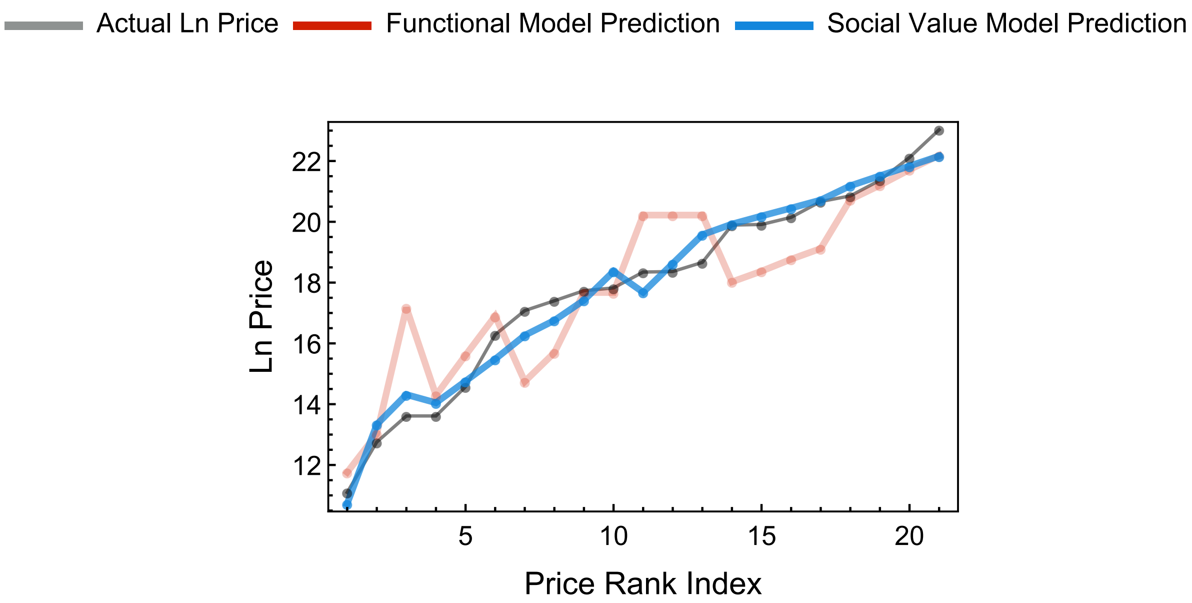

Figure S20: Real and predicted values for the Ballistic Control System module class.

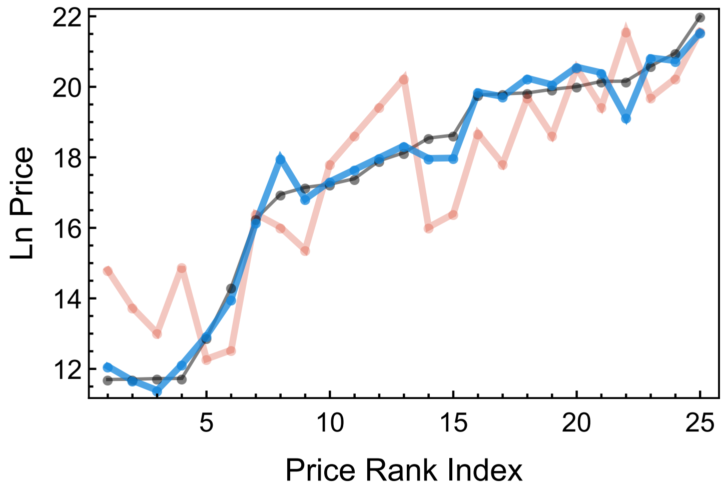

Figure S21: Real and predicted values for the Magnetic Field Stabilizer module class.

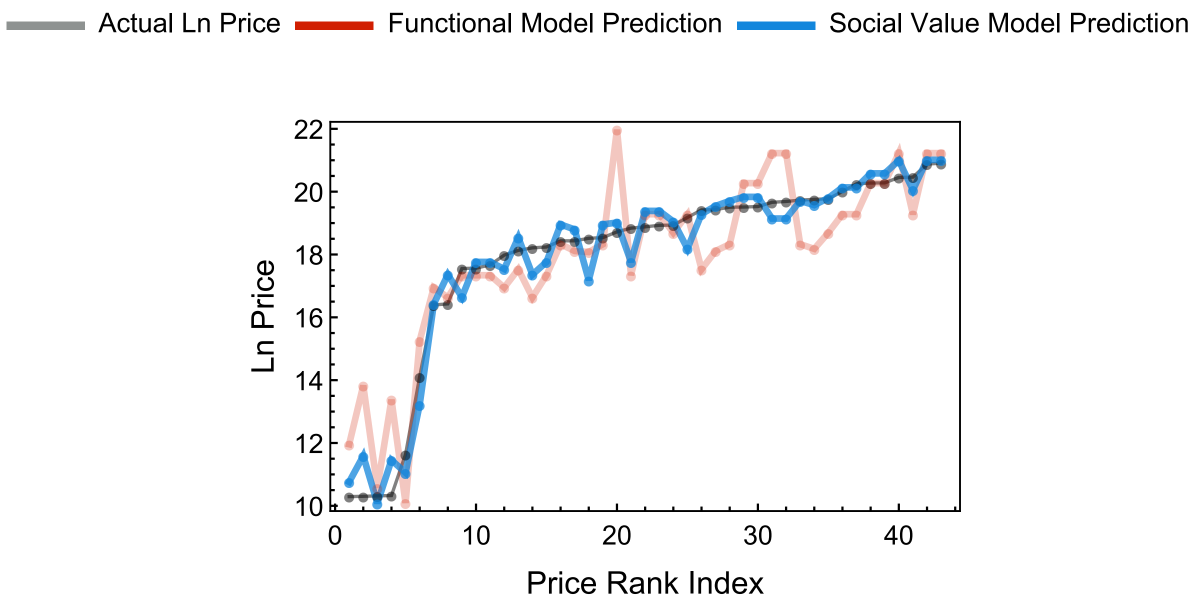

Figure S22: Real and predicted values for the Heat Sink module class.

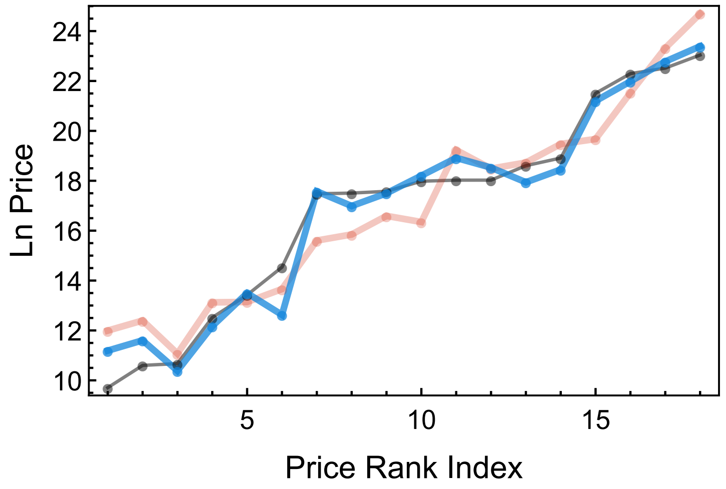

Figure S23: Real and predicted values for the Gyrostabilizer module class.

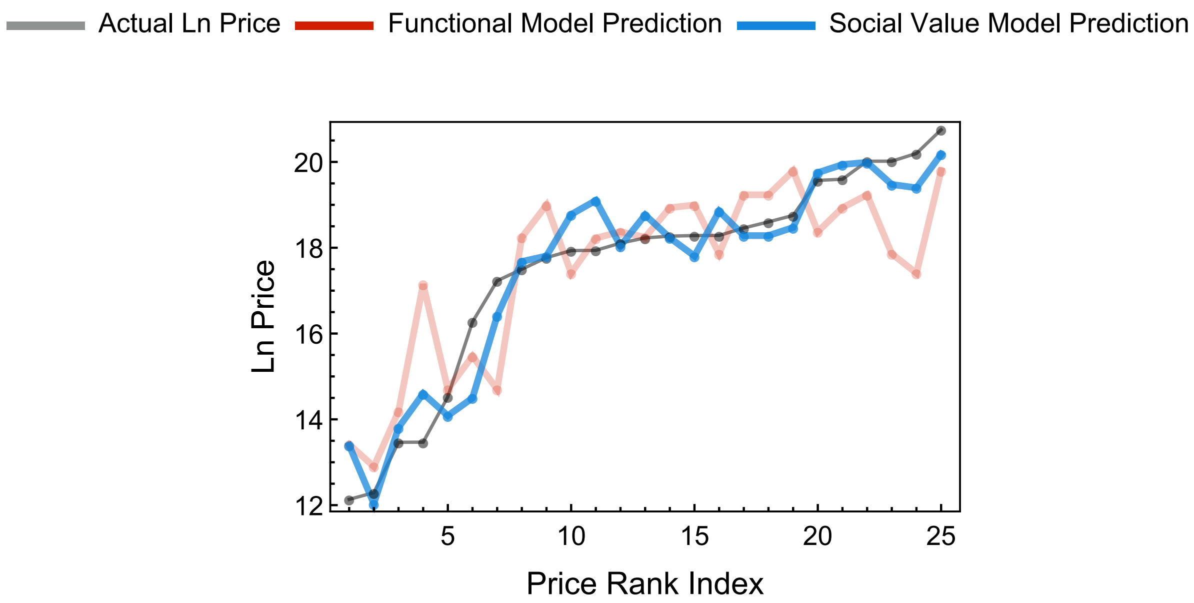

Figure S24: Real and predicted values for the Shield Boost Amplifier module class.

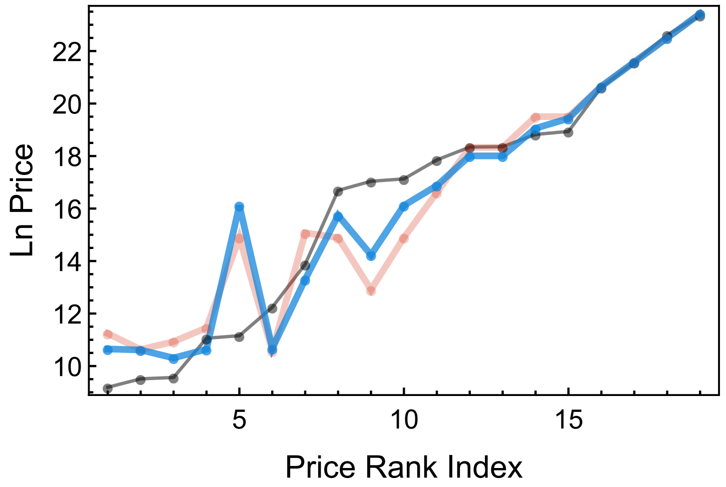

Figure S25: Real and predicted values for the Adaptive Invulnerability Field module class.

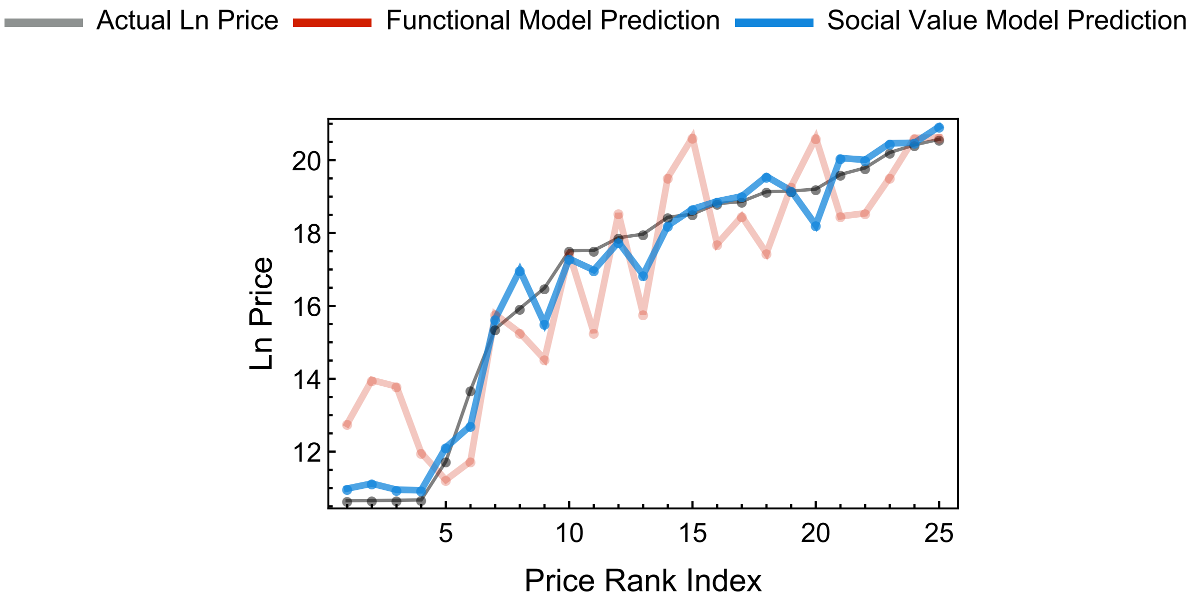

Figure S26: Real and predicted values for the Armor Explosive Hardener module class.

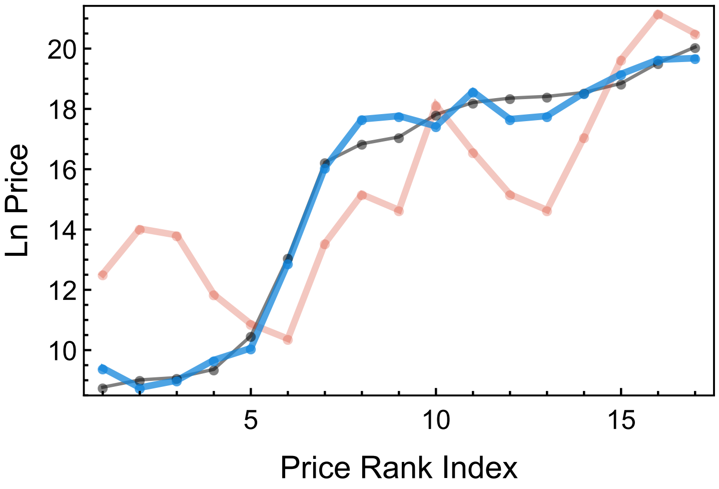

Figure S27: Real and predicted values for the Large Armor Repair Unit module class.

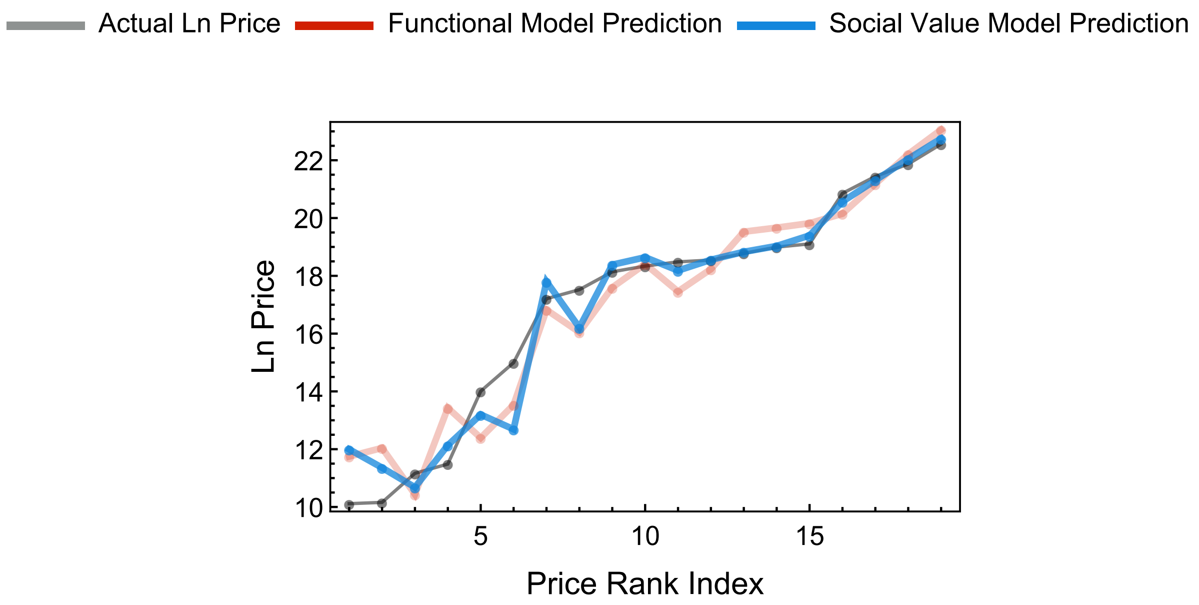

Figure S28: Real and predicted values for the X-Large Shield Booster module class.

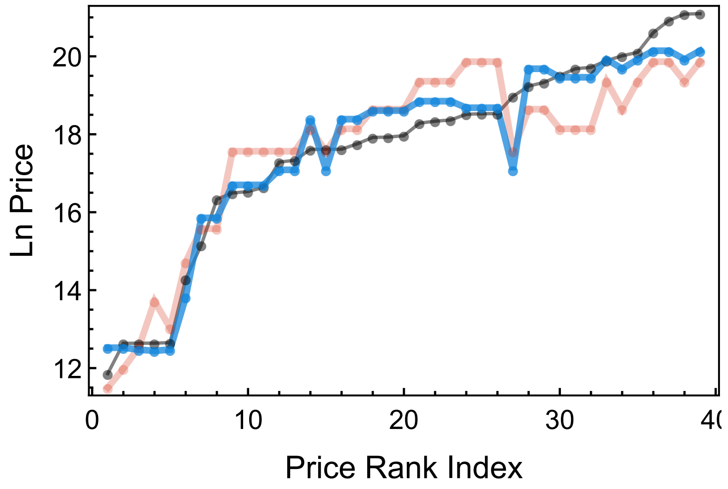

Figure S29: Real and predicted values for the Large Shield Booster module class.

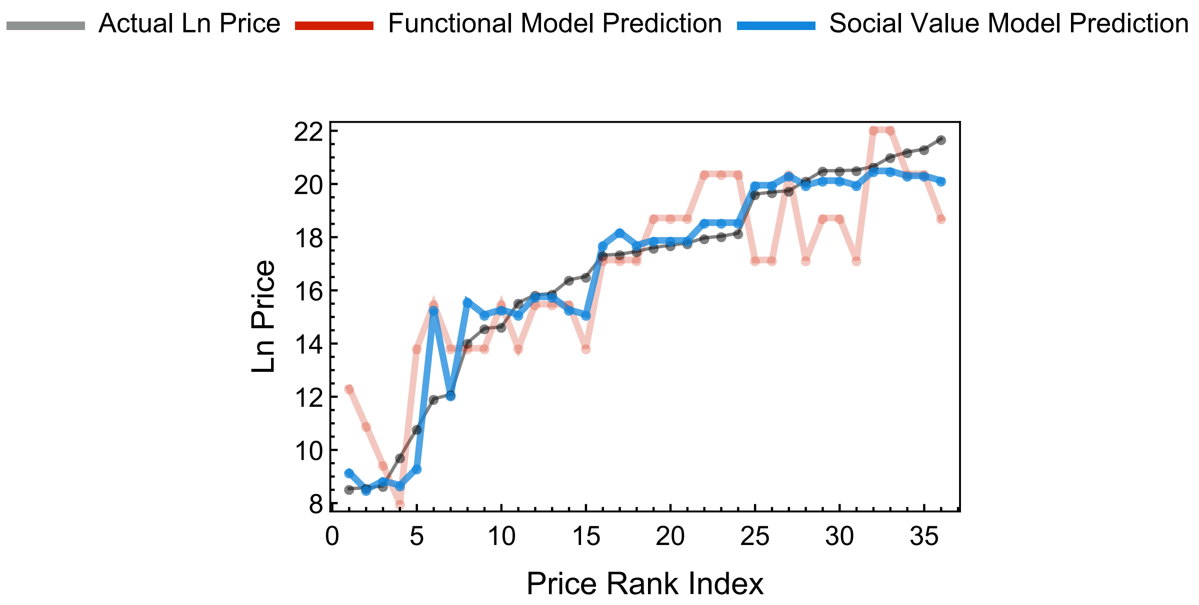

Figure S30: Real and predicted values for the Medium Shield Booster module class.

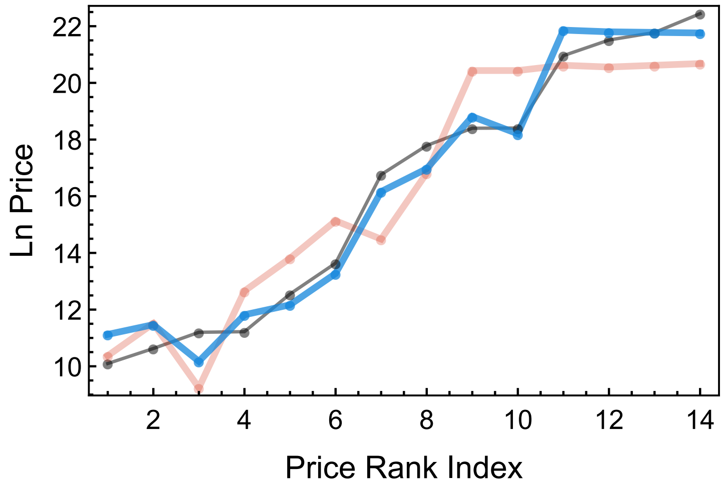

Figure S31: Real and predicted values for the Stasis Webifier module class.

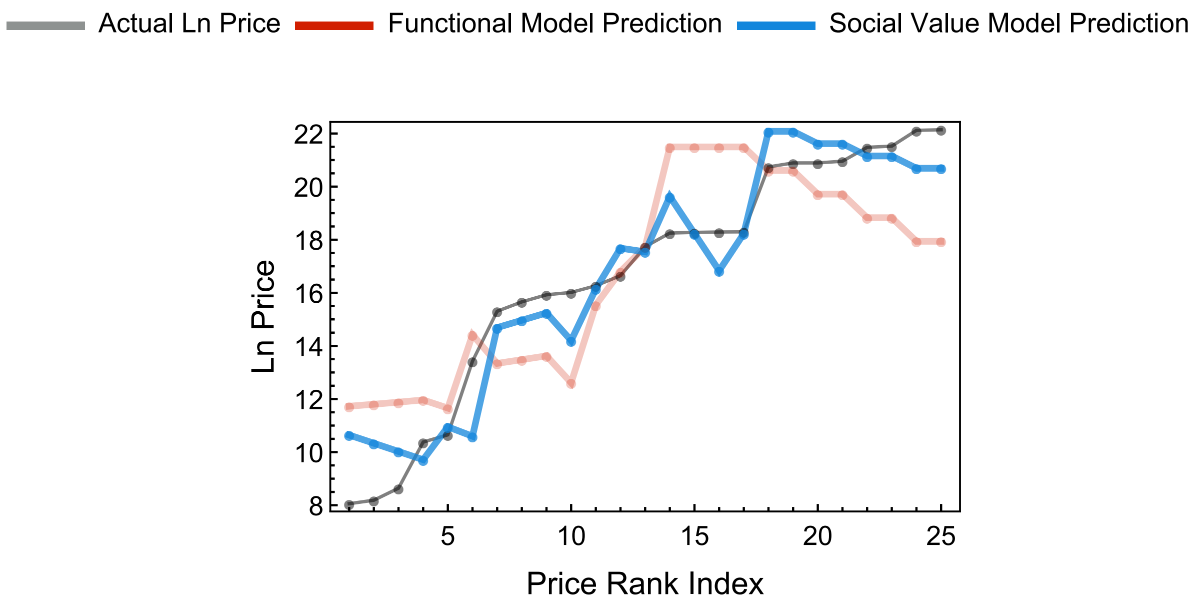

Figure S32: Real and predicted values for the Warp Disruptor module class.

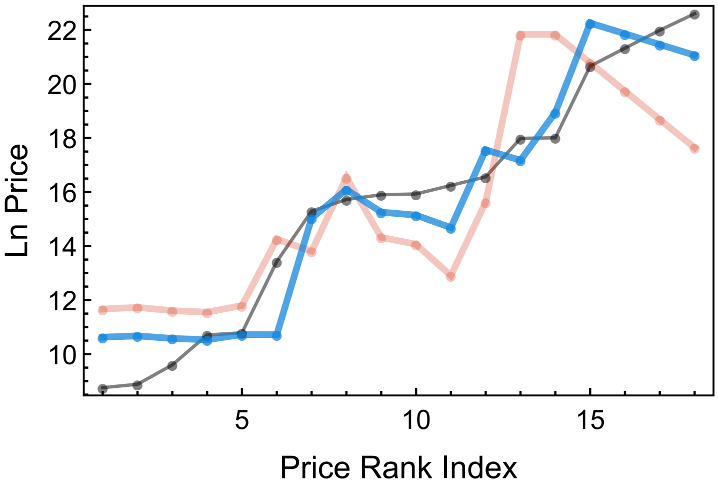

Figure S33: Real and predicted values for the Warp Scrambler module class.

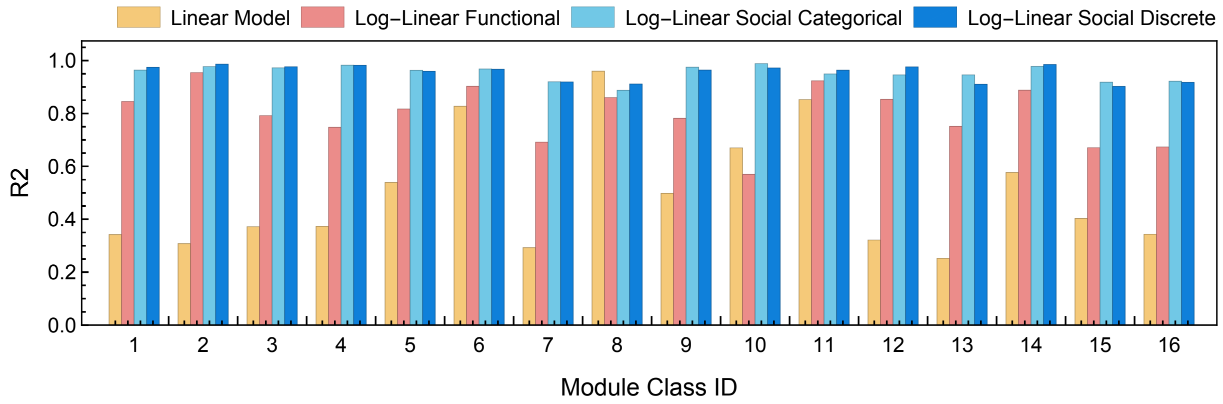

Figure S34: Comparison of the R2 scores across the 16 module classes and our four model types. The two log-linear social models are consistently better than the log-linear functional model, which is typically much better than the linear models (although for class 8 and 10 the linear is better, and for 8 it is the best).

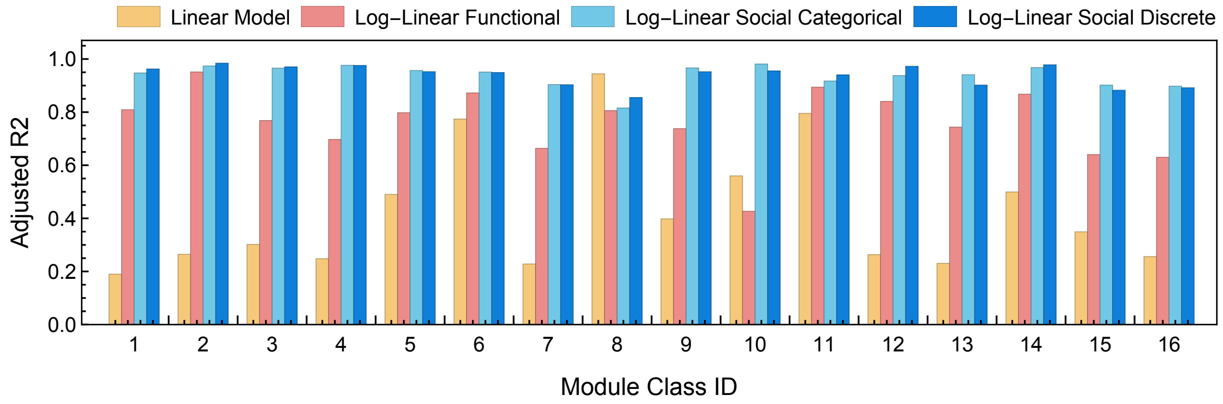

Figure S35: Comparison of the adjusted R2 scores across the 16 module classes and our four model types. The two log-linear social models are consistently better than the log-linear functional model, which is typically much better than the linear models (although for class 8 and 10 the linear is better, and for 8 it is the best).

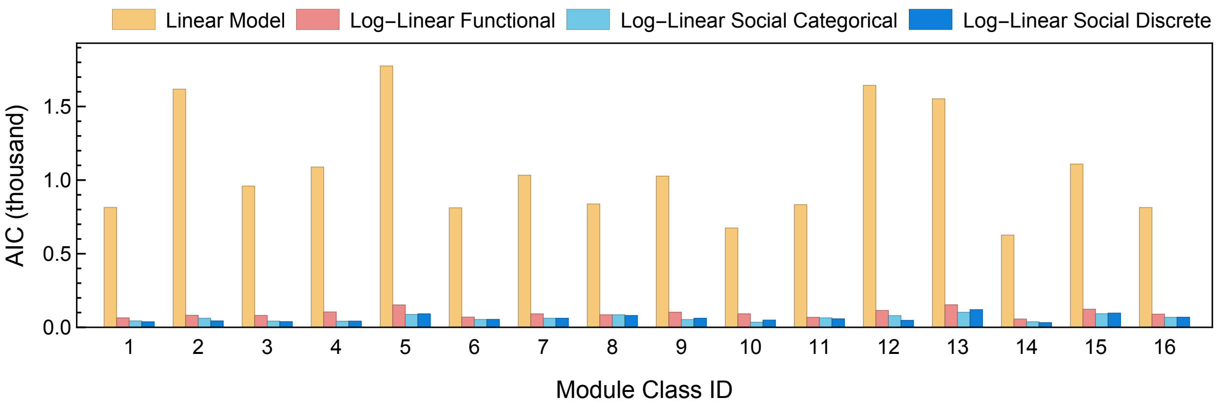

Figure S36: Comparison of the AIC scores across the 16 module classes and our four model types (lower is better). The two log-linear social models are consistently slightly better than the log-linear functional model, which is much better than any linear model.
